# Atmospheric Particulate Matter as a Carrier of Pb, Cd, and Ni: From Environmental Transfer and Bioaccessibility to Molecular Toxicity and Predictive Modeling

**DOI:** 10.3390/jox16050167

**Published:** 2026-09-03

**Authors:** Raluca Grădinaru, Setalia Popa, Ionuț Ciprian Popa, Andrei Cristian Grădinaru, Irina Radinschi, Silviu Gurlui, Liviu Leontie

**Affiliations:** 1 Faculty of Physics, Alexandru Ioan Cuza University of Iasi, 11 Carol I Blvd., 700506 Iasi, Romania; gradinaru.raluca.gr@gmail.com (R.G.); sgurlui@uaic.ro (S.G.); lleontie@uaic.ro (L.L.); 2 Grigore T. Popa University of Medicine and Pharmacy, 700115 Iasi, Romania; setalia.popa@umfiasi.ro; 3 INAF—Osservatorio Astronomico di Capodimonte, Salita Moiariello, 16, 80131 Napoli, Italy; ciprian.popa@inaf.it; 4 “Ion Ionescu de la Brad” Iasi University of Life Sciences, 3 Sadoveanu Alley, 700490 Iasi, Romania; 5 Department of Physics, “Gheorghe Asachi” Technical University, 700050 Iasi, Romania; radinschi@yahoo.com

**Keywords:** particulate matter, lead, cadmium, nickel, bioaccessibility, environmental transfer, food-chain exposure, molecular toxicology, predictive modeling, One Health

## Abstract

Atmospheric particulate matter (PM) is a heterogeneous carrier of toxic metals whose environmental fate and biological effects depend on particle size, source-related composition, chemical form, solubility, and bioaccessibility. Lead (Pb), cadmium (Cd), and nickel (Ni) are of particular concern because atmospheric transport and deposition connect air pollution with persistent contamination of soils, vegetation, waters, sediments, food, and feed, followed by human and animal exposure. This review integrates evidence across a source-to-effect continuum encompassing emission, atmospheric transport, deposition, post-depositional redistribution, food-chain transfer, bioaccessibility, toxicokinetics, molecular toxicity, biomonitoring, remediation, and predictive assessment. Total PM mass and total metal concentration do not adequately represent biologically effective exposure, which is additionally determined by respiratory deposition, gastrointestinal release, dissolution kinetics, absorption, tissue distribution, intracellular retention, and interactions with co-associated constituents. Pb, Cd, and Ni share downstream effects including oxidative imbalance, inflammation, mitochondrial dysfunction, DNA damage, impaired genome maintenance, epigenetic remodeling, and cytogenetic abnormalities, but differ in environmental mobility, persistence, target-organ distribution, and molecular mechanisms. Effective risk assessment therefore requires coordinated multi-matrix monitoring, distinction between total and biologically accessible fractions, pathway-specific remediation, and appropriately validated predictive models. An integrated One Health framework can improve identification of priority matrices, exposure pathways, and risk-reduction measures.

## 1. Introduction

Clean air is essential to human health, yet air pollution remains a major environmental and public-health challenge. The 2021 World Health Organization air-quality guidelines reflect evidence of adverse effects at pollutant concentrations lower than those addressed in earlier guidance [1]. In Europe, air pollution has declined steadily over recent decades but remains the region’s largest environmental health risk [2]. Contemporary air-quality research increasingly integrates emission characterization, atmospheric measurement and modeling, chemical composition, source attribution, exposure assessment, and health-impact evaluation [3].

Atmospheric particulate matter (PM) is a heterogeneous suspension of solid particles and liquid droplets, commonly characterized using operational aerodynamic size fractions. PM_10_ and PM_2.5_ denote operational particulate-matter mass fractions collected with nominal aerodynamic cut points of 10 and 2.5 µm, respectively [1]. Beyond its mass concentration, PM acts as a carrier of chemically and physically diverse inorganic and organic constituents whose environmental behavior and biological relevance depend on particle size, source, composition, surface properties, and atmospheric processing [3]. Among PM-associated metals, lead (Pb), cadmium (Cd), and nickel (Ni) warrant particular attention because they are regulated under the European air-quality framework and represent distinct patterns of environmental persistence, transfer, and toxicity. Under Directive (EU) 2024/2881, the annual limit value for Pb is 0.5 µg/m^3^. Annual target values of 5 ng/m^3^ for Cd and 20 ng/m^3^ for Ni are to be attained by 11 December 2026, while the same numerical concentrations are established as annual limit values to be attained by 1 January 2030 [4].

Atmospheric Pb, Cd, and Ni originate from combinations of metallurgical and other industrial processes, fossil-fuel combustion, waste incineration, traffic-related emissions, mineral and construction dust, and other source categories [5]. Following emission, carrier particles undergo dispersion, dilution, condensation, coagulation, and atmospheric aging, which may modify particle composition and mixing state [3,6]. Aerodynamic size and meteorological conditions influence atmospheric residence and transport [1,3]. Previously deposited material may be returned to the atmosphere through wind erosion, traffic-induced turbulence, and disturbance of contaminated road dust or soil [7,8]. Metal-bearing particles are removed through wet and dry deposition, with efficiencies determined by particle properties, precipitation, and receiving-surface characteristics [9,10].

The biological relevance of PM-associated metals cannot be inferred from atmospheric concentration alone. For inhaled particles, metal release under simulated pulmonary conditions depends on the metal-bearing phase, particle matrix, fluid composition, pH, residence time, and solid-to-liquid ratio [11,12]. Gastrointestinal release after ingestion likewise depends on metal form and matrix characteristics [13]. Bioaccessibility denotes the fraction released under defined conditions and potentially available for absorption, whereas bioavailability requires actual absorption and availability within the organism [11,12,13,14]. Following deposition, metals may undergo sorption–desorption, complexation, precipitation–dissolution, redox reactions, and redistribution among mineral and organic phases [15,16]. Because Pb, Cd, and Ni are elements, they cannot be degraded into innocuous products; their environmental persistence instead reflects redistribution and changes in speciation, solubility, mobility, and biological availability [15,16].

The three metals differ substantially in toxicokinetics, biological persistence, and carcinogenic hazard. Inorganic Pb compounds are classified as probably carcinogenic to humans (Group 2A), whereas Cd and Cd compounds and Ni compounds are classified as carcinogenic to humans (Group 1) [17,18]. No blood Pb concentration without known adverse effects has been identified, and children are particularly susceptible to its neurological and developmental effects [19]. Pb can accumulate in mineralized tissues, where slowly exchanging skeletal pools may retain the metal for years to decades and provide an endogenous source during bone resorption [20]. Cd exhibits pronounced cumulative persistence, with model-derived biological half-times of approximately 4–19 years in the human liver and 6–38 years in the kidney [21]. Systemically absorbed Ni is generally eliminated more rapidly, predominantly through urine, but absorption, respiratory retention, distribution, and biological effects depend strongly on chemical form, compound solubility, particle size, and exposure route [22,23]. Equal atmospheric concentrations of Pb, Cd, and Ni therefore do not imply equivalent internal doses, target-organ burdens, or toxicological outcomes.

Mass-based PM metrics provide only a partial description of metal-bearing particles. PM mass concentration alone does not capture site-to-site differences in elemental composition, source contributions, oxidative activity, or estimated health risk [24,25,26]. Oxidative potential (OP) is an operational measure of the capacity of a PM sample to oxidize target molecules or generate redox-active responses under defined assay conditions [24,25,27]. Its magnitude is assay-dependent and reflects interactions among redox-active metals, organic compounds, carbonaceous material, secondary inorganic components, and other particle constituents; it is not a direct surrogate for total metal concentration [24,25]. Particle mass, elemental concentration, chemical form, bioaccessibility, and OP should therefore be treated as complementary rather than interchangeable descriptors.

Evidence relevant to PM-associated metals is distributed across atmospheric science, environmental chemistry, food-safety assessment, toxicology, biomonitoring, remediation, and quantitative modeling. This review integrates these domains within a source-to-effect framework linking emission and particle association to atmospheric transport, deposition, post-depositional redistribution, food-chain transfer, bioaccessibility, internal dose, molecular and cytogenetic effects, monitoring, remediation, and predictive assessment. Particular emphasis is placed on identifying where total environmental burden becomes biologically effective exposure, how Pb, Cd, and Ni differ along this continuum, and which measurements and interventions are most informative within a One Health perspective.

## 2. Methodological Approach of the Review

### 2.1. Review Design and Scope

This article was developed as an integrative narrative review with a structured, mechanism-oriented synthesis of evidence from atmospheric science, environmental chemistry, exposure assessment, food-chain transfer, toxicokinetics, molecular toxicology, biomonitoring, predictive modeling, and remediation. Its objective was to examine particulate-matter-associated Pb, Cd, and Ni across a continuous source-to-effect framework linking emission and particle association with atmospheric transport, deposition, post-depositional redistribution, biological accessibility, internal exposure, molecular toxicity, monitoring, prediction, and intervention.

A narrative rather than meta-analytical design was selected because the available evidence encompasses heterogeneous environmental matrices, particle-size fractions, sampling and analytical procedures, exposure scenarios, biological models, molecular endpoints, and regulatory contexts. The synthesis therefore prioritized mechanistic coherence, metal- and matrix-specific interpretation, cross-compartment transfer processes, methodological limitations, and the distinction among total concentration, bioaccessible fractions, absorbed dose, and biological effect.

The scope encompassed atmospheric sources and particle association; transport, physicochemical transformation, deposition, and resuspension; terrestrial and aquatic redistribution; soil–plant, feed–animal, and food-chain transfer; bioaccessibility and bioavailability; human and animal exposure; toxicokinetics; oxidative, inflammatory, mitochondrial, genotoxic, cytogenetic, and epigenetic effects; biomonitoring; predictive modeling; and remediation.

Pb, Cd, and Ni were selected for comparative evaluation because all three are regulated as atmospheric metal pollutants within the European air-quality framework and are commonly associated with atmospheric particulate matter, while differing markedly in dominant emission sources, particle association, environmental mobility, chemical form, biological persistence, toxicokinetics, target-organ distribution, and carcinogenic mechanisms. Their parallel assessment therefore provides a comparative model for distinguishing shared particle-mediated exposure processes from metal-specific determinants of environmental and biological risk.

### 2.2. Information Sources, Search Strategy, and Evidence Selection

Targeted literature searches were conducted using PubMed, Web of Science, Scopus, ScienceDirect, and Google Scholar, with the final literature update performed on 4 July 2026. The evidence base included peer-reviewed primary studies, authoritative reviews, regulatory documents, toxicological profiles, technical reports, and institutional assessments. Official information was obtained from the World Health Organization (WHO), European Environment Agency (EEA), European Union (EU) and EUR-Lex, International Agency for Research on Cancer (IARC), European Food Safety Authority (EFSA), Food and Agriculture Organization of the United Nations (FAO), Joint FAO/WHO Expert Committee on Food Additives (JECFA), Agency for Toxic Substances and Disease Registry (ATSDR), and relevant national public-health and environmental agencies.

Searches used combinations of the terms lead, cadmium, nickel, Pb, Cd, and Ni with terms related to particulate matter, particle size, atmospheric transport and deposition, environmental transfer, food-chain contamination, chemical speciation, solubility, bioaccessibility, bioavailability, internal dose, toxicokinetics, oxidative stress, inflammation, mitochondrial dysfunction, DNA damage and repair, cytogenetic damage and chromosome instability, epigenetic regulation, carcinogenicity, biomonitoring, predictive modeling, and remediation. Search terms and combinations were adapted to the indexing and search functions of each information source. Terminology was iteratively expanded using synonymous expressions, specific chemical forms, pathway-related terms, and examination of the reference lists of relevant primary studies and reviews. Because the review was designed as an integrative narrative synthesis rather than a systematic review, literature identification followed a targeted and iterative approach rather than a single predefined search string and formal study-screening workflow. Searches were refined according to the specific environmental compartment, exposure pathway, metal, chemical form, biological endpoint, or modeling application under consideration. Consequently, the evidence base was assembled to support mechanistic and cross-compartment synthesis rather than exhaustive enumeration of all records retrieved from each database.

Sources were selected according to their direct relevance to one or more stages of the source-to-effect continuum, methodological clarity, metal- and matrix-specific applicability, and capacity to support mechanistic, quantitative, analytical, or regulatory interpretation. Primary studies were preferentially used for specific experimental, analytical, or quantitative statements, whereas authoritative reviews and institutional assessments were used for broader conceptual frameworks, toxicokinetic interpretation, hazard classification, and regulatory context. Accordingly, the relative contribution of different evidence types varied among sections: atmospheric and environmental-transfer sections relied predominantly on field, analytical, and modeling studies; toxicological sections incorporated experimental and biomonitoring evidence together with authoritative toxicological assessments; and regulatory sections relied primarily on official institutional and legislative sources. Evidence obtained for different metals, chemical forms, environmental matrices, species, exposure routes, or biological endpoints was not considered automatically interchangeable.

No quantitative pooling or formal study-level risk-of-bias assessment was undertaken because the review integrated diverse evidence types and was not designed to estimate a common effect size. Instead, the evidence was critically interpreted according to study design, analytical approach, matrix and exposure relevance, chemical form, biological model, endpoint specificity, and consistency with independent experimental or authoritative evidence.

The multidisciplinary scope of this review introduces heterogeneity in terminology, sampling strategies, particle-size definitions, analytical protocols, units of measurement, exposure models, biological systems, and reported endpoints. Consequently, quantitative values were interpreted as context-specific unless supported across multiple studies or by authoritative assessments, and direct extrapolation among different metals, chemical forms, matrices, exposure routes, species, tissues, or experimental conditions was avoided. Regulatory information was evaluated using official sources available at the time of the final literature update.

### 2.3. Conceptual Framework for Evidence Synthesis

The evidence was organized into four interconnected analytical pillars:

Pillar I: Atmospheric source-to-particle processes, encompassing natural and anthropogenic sources, emission characteristics, particle-size distribution, metal–particle association, atmospheric aging, transport, deposition, resuspension, and source attribution.

Pillar II: Environmental transfer and biological accessibility, addressing redistribution through soils, vegetation, surface waters, sediments, agricultural systems, animal feed, and food products, with emphasis on chemical speciation, solubility, bioaccessibility, bioavailability, and transfer across environmental and biological interfaces.

Pillar III: Internal dose and molecular toxicity, integrating exposure pathways, absorption, toxicokinetics, tissue distribution, biological persistence, oxidative imbalance, inflammation, mitochondrial dysfunction, DNA damage, repair impairment, cytogenetic abnormalities, epigenetic remodeling, and carcinogenic mechanisms.

Pillar IV: Monitoring, prediction, and intervention, connecting analytical characterization, bioaccessibility testing, biomonitoring, sentinel organisms, transfer metrics, statistical and spatial modeling, uncertainty assessment, machine-learning approaches, and remediation strategies.

These pillars were interpreted as interconnected components of a source-to-effect continuum rather than as independent thematic categories. The synthesis distinguished direct empirical evidence from mechanistic inference and metal-specific findings from effects attributed to the complete particulate mixture. Predictive models were evaluated according to their capacity to connect clearly defined stages of the continuum and to support monitoring, prioritization, or intervention, rather than as substitutes for mechanistic evidence or direct environmental and biological measurements.

## 3. Environmental Sources and Metal-Specific Characteristics of Pb, Cd, and Ni

Atmospheric Pb, Cd, and Ni originate from both natural and anthropogenic processes, but their relative contributions and environmental behavior differ according to emission history, source technology, geological setting, chemical form, and particle-generation mechanism [5,28,29,30,31]. Pb remains strongly influenced by legacy contamination originating from past anthropogenic emissions, including historical use of leaded petrol and industrial releases, and by the subsequent resuspension of contaminated surface reservoirs [7,8]. Cd is closely associated with non-ferrous metallurgy and other high-temperature industrial processes [5,21,30,31], whereas Ni reflects a combination of geogenic inputs, fuel combustion, and industrial activities, with environmental mobility and biological accessibility strongly influenced by chemical form and compound solubility [5,22,23]. Source interpretation therefore requires a metal-specific approach that integrates emission context, particle-size distribution, chemical form, elemental co-occurrence, geological background, and local environmental conditions.

### 3.1. Natural and Anthropogenic Contributions to Atmospheric Metal Burdens

Natural atmospheric inputs of trace metals include wind-driven entrainment of soil and mineral particles, volcanic emissions, wildland fires, sea-salt spray, meteoric material, and biogenic particles [28,29]. Estimates of their global magnitude vary considerably because inventories differ in source coverage, emission factors, spatial resolution, and treatment of temporal and environmental variability [28,29]. Natural background contributions should therefore be interpreted as model-dependent estimates rather than fixed global values.

Anthropogenic atmospheric inputs arise primarily from non-ferrous metallurgy, mining and ore processing, coal and oil combustion, waste incineration, iron and steel production, industrial manufacturing, traffic-related activities, and mechanically generated dust [5,30,31]. Previously deposited metals may also be returned to the atmosphere through wind erosion, traffic-induced turbulence, construction, and disturbance of contaminated soils, road dust, or industrial surface materials [7,8]. The relative importance of these sources varies with raw-material composition, fuel use, industrial structure, process technology, emission-control efficiency, and historical contamination.

Elemental co-occurrence patterns can assist source interpretation but should not be treated as unique fingerprints. Co-occurrence of Ni and vanadium (V) may support attribution to residual-fuel-oil combustion, particularly in marine-engine emissions [32]. Cd enrichment, particularly in fine particulate fractions, may support attribution to non-ferrous metallurgy, smelting, waste incineration, and other high-temperature industrial processes when interpreted together with co-occurring elements and local source information [21,30,31,33,34]. Because Cd may also occur in mechanically generated industrial dust and resuspended contaminated material, its presence alone should not be considered a unique source tracer [21,30,31]. Enrichment in copper (Cu), antimony (Sb), and barium (Ba) may indicate non-exhaust traffic emissions, whereas aluminum (Al), calcium (Ca), and iron (Fe) are commonly associated with crustal material or resuspended mineral dust [8,35]. Elevated Pb in urban dust may reflect the remobilization of historically contaminated soils and surface deposits [7]. Such associations should be evaluated together with particle-size distributions, temporal patterns, meteorological conditions, land use, and independent information on local and regional emission sources.

Positive matrix factorization (PMF) is widely used for receptor-based source apportionment, but the resulting factors do not automatically represent uniquely identified emission sources. Their interpretation requires integration with source tracers, temporal and meteorological information, emission inventories, particle-size distributions, and documented local activities [35,36,37].

The principal atmospheric sources and source-related characteristics of Pb, Cd, and Ni are summarized comparatively in Table 1, providing an overview of their major anthropogenic and geogenic contributions, PM-associated characteristics, source indicators, and regional considerations relevant to source attribution. 

### 3.2. Lead Sources, Chemical Forms, and Legacy Pathways

Pb occurs predominantly as Pb(II) in environmental systems, whereas Pb(IV) is comparatively uncommon [20]. Galena (PbS) is the principal Pb sulfide ore mineral. Its oxidative weathering can release Pb and promote its redistribution into secondary mineral phases; under neutral mine-waste conditions, Pb released from galena oxidation has been shown to accumulate in secondary carbonate phases, including cerussite (PbCO_3_) [43]. Mineralogical form is therefore an important determinant of Pb dissolution, mobility, and persistence.

Naturally occurring Pb comprises four stable isotopes: ^204^Pb, ^206^Pb, ^207^Pb, and ^208^Pb. The latter three contain radiogenic contributions from the uranium and thorium decay series. Ratios such as ^206^Pb/^207^Pb and ^208^Pb/^206^Pb can assist source discrimination and mixing analysis when the potential end-members have sufficiently distinct isotopic compositions [42]. Isotopic evidence is most informative when combined with Pb concentrations, spatial patterns, emission history, and independent source information [42].

Atmospheric Pb is predominantly particle-associated and may occur in metallurgical aerosols, combustion-derived particles, industrial and mineral dust, and resuspended contaminated soil or road dust. Contemporary atmospheric burdens may consequently represent a mixture of ongoing primary emissions and Pb deposited during previous periods of anthropogenic activity, including leaded-petrol use and industrial emissions, that is subsequently reintroduced into the atmosphere through resuspension [5,7,8].

The global phase-out of leaded petrol for road vehicles was completed in 2021, when Algeria discontinued its use [44]. Although this transition eliminated leaded petrol for road vehicles as a major global primary-emission source, it did not remove Pb accumulated during previous decades in roadside soils, urban dust, sediments, and other environmental reservoirs [7,44]. In contaminated environments, these reservoirs may continue to contribute to atmospheric Pb through erosion and resuspension [7].

Current Pb sources are region-specific and include mining and ore processing, primary and secondary metal production, smelting, lead–acid battery manufacture and recycling, combustion processes, waste handling, and disturbance of contaminated surface materials [5,30,31]. In the United States, aircraft operating with leaded aviation gasoline, primarily small piston-engine aircraft, remain an important atmospheric source, particularly in the vicinity of affected airports [38]. This source profile should not be extrapolated automatically to regions with different aviation-fuel use, industrial activities, and regulatory histories; the relative contribution of Pb sources should therefore be interpreted within the regional emission and regulatory context.

Following deposition, Pb is generally retained strongly in surface soils through sorption, complexation, ion exchange, and precipitation processes [15,16]. Retention depends on pH, mineralogy, iron (Fe) and manganese (Mn) (oxyhydr)oxides, organic matter, competing ions, and redox conditions, whereas acidification may increase Pb dissolution and mobility [15,16]. This persistence is particularly relevant to PM-associated Pb because contaminated near-surface soils and dust can act as long-term reservoirs available for subsequent erosion and atmospheric resuspension [7,20].

### 3.3. Cadmium Sources, High-Temperature Redistribution, and Environmental Mobility

Cd is a comparatively scarce element that occurs mainly in association with zinc ores and complex polymetallic deposits [21]. Its production is closely linked to the processing of non-ferrous ores, particularly zinc-containing materials, with additional recovery from Pb and Cu processing and from the recycling of Cd-containing products [21]. In environmental systems, Cd occurs predominantly as Cd(II) and may be present as hydrated ions, inorganic or organic complexes, sorbed or exchangeable forms, and sparingly soluble precipitates [15,16,21].

Major atmospheric sources include non-ferrous smelting and refining, fossil-fuel combustion, waste incineration, and other high-temperature industrial processes [5,21,30,31]. Local emissions may also arise from the manufacture, processing, recycling, disposal, or mechanical disturbance of Cd-containing materials and contaminated industrial dust [5,21].

High-temperature redistribution is an important characteristic of industrial Cd emissions. During smelting, combustion, or incineration, Cd may be released in vapor and particulate forms. As exhaust gases cool, vapor-phase Cd can condense, adsorb, or react on particle surfaces, favoring its association with fine or respirable particles under some process conditions [5,21,30,31]. This behavior represents temperature-dependent phase redistribution and should not be interpreted as evidence that Cd is intrinsically volatile under ordinary environmental conditions.

The presence of Cd in fine particulate matter is consequently a useful tendency but not an exclusive source indicator. Mechanically generated industrial dust, contaminated surface materials, atmospheric mixing, particle agglomeration, and resuspension can distribute Cd across several aerodynamic fractions [5,21,30,31]. Size-resolved Cd concentrations should therefore be interpreted together with source context, particle composition, elemental co-occurrence, and meteorological conditions.

Following deposition, Cd is generally more mobile than Pb in soils, particularly under acidic conditions or where sorption capacity is limited [15,16,21]. Its retention and mobility are controlled by sorption–desorption, ion exchange, complexation, precipitation–dissolution reactions, organic matter, mineralogy, competing ions, and redox conditions; acidification generally promotes Cd desorption, dissolution, and leaching, whereas sorption and formation of less soluble phases can reduce mobility [15,16]. These post-depositional processes are relevant to PM-associated Cd because they determine whether deposited Cd remains in surface reservoirs, becomes available for biological uptake, or is redistributed through dissolved, particulate, or resuspended pathways.

### 3.4. Nickel Sources, Chemical Forms, and Fuel-Related Signatures

Ni is naturally distributed in the Earth’s crust and occurs in economically important magmatic sulfide and lateritic deposits [23,40,45]. Ni-rich lateritic regolith develops through prolonged weathering of ultramafic parent rocks, whereas magmatic sulfide deposits represent a distinct geological source [40,45]. Weathering and erosion of Ni-rich rocks and soils can create elevated geogenic backgrounds and introduce Ni-bearing mineral particles into the atmosphere [23,40].

Atmospheric Ni occurs in chemically diverse forms that reflect geological origin, fuel composition, and industrial process. Nickel chloride and nickel sulfate are water-soluble, whereas nickel oxide, nickel sulfide, and nickel subsulfide are poorly water-soluble [23]. Nickel carbonyl is a volatile organometallic compound with specialized industrial relevance and severe acute inhalation toxicity [23]. In polluted air, the principal Ni forms may include sulfate, oxides, sulfides, and smaller contributions from elemental Ni [23].

This chemical diversity is environmentally and toxicologically important. Aerodynamic size influences respiratory deposition, whereas compound solubility and dissolution kinetics affect particle persistence, clearance, local tissue availability, systemic absorption, and bioaccessibility [11,12,23]. Chemical form and particle size should therefore be characterized independently rather than inferred from total Ni concentration.

Natural atmospheric sources include windblown mineral dust, volcanic emissions, vegetation-derived particles, wildland fires, and meteoric material [23,28,29]. Major anthropogenic sources include coal and oil combustion, non-ferrous metal processing, Ni refining, steel and alloy manufacture, electroplating, and waste incineration [5,23,30,31].

Residual-fuel combustion is particularly relevant to source attribution because Ni and V commonly occur together in heavy-fuel-oil emissions, including emissions from marine engines [32,39]. Co-occurrence of Ni and V in fine particulate matter, together with characteristic V/Ni ratios, may support identification of residual-oil or shipping-related contributions when interpreted alongside additional tracers and local fuel-use information [32,39]. However, Ni–V associations and V/Ni ratios should be regarded as context-dependent source indicators rather than unique fingerprints, because both elements may also originate from other combustion and industrial sources, and their relative abundance varies with fuel composition, combustion technology, geographical setting, and time [30,31,39]. Ni is not source-exclusive and may also originate from coal combustion, metallurgy, alloy production, industrial dust, and geogenic mineral material [5,23,28,29,30,31].

Fine-particle Ni is often associated with combustion-related emissions, particularly oil combustion, whereas coarse-fraction Ni may reflect mineral dust, road dust, abrasion, resuspension, or mixed urban and industrial sources [8,32,35,46]. These tendencies are site-specific. No single aerodynamic fraction can be assigned universally to Ni, and size-resolved results should be interpreted together with elemental co-occurrence, local activities, receptor-model outputs, and independent evidence regarding emission sources [35,37,46]. Compound solubility should be evaluated separately because it primarily informs dissolution, persistence, bioaccessibility, and potential bioavailability [11,12,23].

Following deposition, Ni mobility in soils is governed by pH, mineralogy, surface charge, cation-exchange capacity, Fe and Mn (oxyhydr)oxides, organic matter, ligand complexation, and competing cations [15,16]. Acidification may promote Ni desorption and dissolved transport, whereas solid organic matter and mineral surfaces can favor retention; conversely, dissolved organic carbon may enhance transport through the formation of soluble Ni–organic complexes [15,16,47]. For PM-associated Ni, these post-depositional processes influence whether deposited Ni remains retained in surface reservoirs or becomes mobile and potentially available for subsequent environmental and biological transfer.

Geological background must be considered explicitly when interpreting Ni concentrations in soil and deposited dust. For example, national-scale soil mapping in the conterminous United States has shown that spatial Ni distributions may be strongly controlled by parent material, with elevated concentrations associated particularly with mafic or ultramafic rocks and shale-derived materials [41]. This regional example illustrates the need to distinguish geogenic background from anthropogenic contributions when interpreting Ni in deposited dust and surface reservoirs. Total Ni concentration alone is therefore insufficient to distinguish geogenic from anthropogenic contributions. Source attribution should integrate geological setting, spatial distribution, particle-size patterns, mineralogical and chemical form, elemental co-occurrence, receptor-model results, and documented local emissions [23,35,37,39,40,41]. Compound solubility should be evaluated separately because it primarily informs dissolution, mobility, bioaccessibility, and potential bioavailability rather than source identity [11,12,15,16,23].

## 4. Atmospheric Particulate Matter as a Carrier of Pb, Cd, and Ni

The environmental and toxicological behavior of particulate-matter-associated Pb, Cd, and Ni reflects the interaction between metal-specific properties and the physicochemical characteristics of their carrier particles. Atmospheric PM comprises heterogeneous particle populations that differ in aerodynamic size, source-related composition, morphology, surface properties, mixing state, metal-bearing phases, solubility, and oxidative activity [3,5,6,11,12,24,25]. These characteristics influence atmospheric residence and transport, wet and dry deposition, post-depositional mobility, respiratory deposition and clearance, metal dissolution, bioaccessibility, and ultimately the relationship between external concentration and biologically effective exposure [1,9,10,11,12,15,16,23,48]. Consequently, equal atmospheric concentrations of Pb, Cd, or Ni do not necessarily represent equivalent environmental mobility, internal exposure, or toxicological relevance.

### 4.1. Particle-Size Fractions and Physicochemical Properties

Aerodynamic diameter is an operational descriptor used to define particle-size fractions and influences atmospheric transport, removal, and respiratory penetration [1,3,9,10]. PM_10_ and PM_2.5_ are operationally defined mass fractions collected using size-selective samplers with nominal aerodynamic cut points of 10 and 2.5 µm, respectively. The coarse particle fraction between the nominal 2.5 and 10 µm cut points (PM_2.5–10_) comprises particles collected between the corresponding nominal cut points [1,46,49]. These fractions are operational categories rather than chemically uniform particle classes, and their measured composition depends partly on sampler design, collection efficiency, sampling location, and analytical procedure [46,49].

Fine and coarse PM commonly differ in their generation mechanisms, atmospheric behavior, and source contributions. Fine particles are frequently associated with combustion emissions, high-temperature industrial processes, and secondary aerosol formation, whereas coarse particles are generally influenced more strongly by crustal material, road dust, mechanical abrasion, sea salt, and resuspension [35,46,49]. These associations are not exclusive. Combustion and industrial sources can generate particles across several size ranges, while mechanical processes may also contribute particles within PM_2.5_. Particle size should therefore be interpreted together with chemical composition, elemental co-occurrence, source-apportionment results, meteorological conditions, and local emission information.

The long-term Boston study provides a well-characterized example of differences between fine and coarse PM [46]. Daily integrated fine and coarse samples collected over nine years showed that black carbon, sulfur, and Pb were associated exclusively with the fine mode, while 84% of vanadium and 79% of Ni occurred in PM_2.5_; by contrast, more than 80% of Ca, Mn, and chlorine occurred in the coarse mode [46]. Source-apportionment analysis further demonstrated distinct source profiles between the fine and coarse fractions, with regional pollution, motor vehicles, wood burning, and oil combustion contributing predominantly to fine PM, whereas crustal and road dust dominated the coarse fraction [46]. These findings demonstrate that similar mass-based PM metrics do not necessarily imply similar elemental composition or source profiles. However, the reported distributions characterize the investigated Boston site and period and should not be treated as universal partitioning coefficients.

A roadside study in Hong Kong similarly showed that PM_2.5_ and PM_2.5–10_ had distinct chemical compositions and source contributions, reflecting different combinations of vehicle emissions, secondary inorganic aerosol, combustion sources, marine aerosol, industrial emissions, and resuspended road dust [49]. Collectively, these studies show that operational size fractions provide important source and transport information but cannot independently identify the origin or chemical form of a metal.

PM samples with comparable mass concentrations may differ markedly in elemental composition, carbonaceous and inorganic constituents, mineral phases, metal–particle associations, mixing state, solubility, and biological reactivity [3,5,6,11,12,24,25]. The same total concentration of Pb, Cd, or Ni may therefore occur within chemically and physically distinct particle matrices and produce different dissolution behavior, environmental mobility, bioaccessibility, and toxicological responses.

Source-related composition contributes substantially to this heterogeneity. Farahani et al. [24] compared PM collected from environments influenced by traffic, biomass burning, secondary aerosol formation, refinery activity, industrial emissions, and dust. The samples differed considerably in organic composition, inorganic ions, elemental content, and mass-normalized oxidative activity. Biomass-burning- and secondary-aerosol-influenced samples exhibited particularly high mass-normalized oxidative activity, whereas total elemental or metal loading alone did not explain the observed differences consistently [24]. These results indicate that PM-associated metals may occur within carbon-rich combustion particles, mineral dust, secondary aerosol, industrial particles, or complex mixtures in which the surrounding matrix modifies their chemical and biological behavior.

Total elemental concentration quantifies the overall burden of a metal in a PM sample, whereas bioaccessibility testing estimates the fraction released under defined experimental or physiologically relevant conditions [11,12]. Bioaccessibility is therefore an operationally defined property rather than an intrinsic constant. Measured values depend on the chemical and mineralogical form of the metal, particle matrix, simulated exposure scenario, extraction-fluid composition and pH, solid-to-liquid ratio, agitation procedure, temperature, and residence time [11,12]. Results obtained using different simulated biological fluids or substantially different protocols should not be considered directly interchangeable.

Bioaccessibility should also be distinguished from bioavailability. A metal released from a particle under simulated physiological conditions may be potentially available for absorption, but the extraction procedure does not directly measure epithelial uptake, systemic absorption, tissue distribution, intracellular dose, or toxicity. Experimental bioaccessibility provides an exposure-relevant operational endpoint between total external concentration and in vivo bioavailability and is most informative when the relevance and analytical performance of the protocol have been demonstrated for the investigated metal, matrix, and exposure pathway [11,12].

Chemical form is particularly important for Pb, Cd, and Ni. Pb may occur in sorbed or surface-associated forms and in phosphate-, oxide-, sulfate-, carbonate-, or sulfide-bearing phases [12,20,43]. Cd may occur in fly ash, mineral phases, or surface-associated forms generated during thermal processes, while its subsequent release depends on speciation and the physicochemical properties of the surrounding particle matrix [11,12,33,34]. Ni compounds range from relatively soluble chlorides and sulfates to poorly soluble oxides, sulfides, and subsulfides [23]. Atmospheric aging may further alter the matrix surrounding these phases through interaction with carbonaceous material, mineral dust, sea salt, secondary inorganic aerosols, and condensed organic constituents [3,5,6,24]. Total concentration and bioaccessible concentration should therefore be treated as complementary, rather than interchangeable, exposure metrics.

OP provides a functional measure of the capacity of a PM sample to oxidize target molecules or generate redox-active responses under defined assay conditions [24,25]. It complements PM mass and chemical-composition measurements but is inherently assay-dependent. Different assays respond to different redox-active constituents and reaction mechanisms, and their results cannot always be compared directly.

In the Swiss multi-site study of Grange et al. [25], road-traffic and wood-combustion sources showed the greatest average mass-normalized oxidative potency among the identified sources, although their relative importance differed between assays and particle-size fractions. Metals associated with non-exhaust traffic emissions, including Cu, Zn, Fe, Sn, Sb, Mn, and Cd, together with tracers of wood combustion, were repeatedly identified as important predictors of volume-normalized OP [25]. The best-performing models commonly combined a traffic-related metal with a wood-combustion tracer. These statistical relationships identify source-related compositional profiles associated with OP but do not demonstrate that the total concentration of any individual metal independently determines particle oxidative activity.

Overall, intrinsic oxidative activity does not show a simple relationship with total elemental or metal content [24,25]. Organic fractions, secondary aerosol components, source-specific mixtures, metal chemical form, solubility, and interactions among particle constituents may influence the measured response [11,12,24,25]. OP should therefore be interpreted as a property of the complete particle mixture under a defined assay system rather than as a direct surrogate for Pb, Cd, Ni, or total-metal concentration [24,25].

### 4.2. Metal–Particle Association and Source-Related Partitioning

Pb, Cd, and Ni may become associated with atmospheric PM through several complementary pathways: (i) direct emission of metal-bearing primary particles; (ii) high-temperature release followed by condensation, adsorption, or chemical reaction on particle surfaces; (iii) mechanical generation and resuspension of metal-containing material; and (iv) post-emission modification of particle composition and mixing state during atmospheric transport.

(i) Direct primary emissions arise when metals are released within particulate material generated by combustion, metallurgy, mineral processing, waste incineration, cement production, and related industrial activities [5,30,31,32,33]. Depending on the source and process, metals may occur in fly ash, soot-associated material, process dust, mineral fragments, or other primary particles. Their concentration and particle association depend on the metal content and mode of occurrence in the fuel or raw material, processing temperature, combustion conditions, particle-formation mechanisms, flue-gas chemistry, and emission-control efficiency [31,32,33].

(ii) High-temperature phase redistribution is particularly important for Cd. During coal combustion, waste incineration, smelting, or other thermal processes, a fraction of Cd may enter the vapor phase. As the exhaust cools, vapor-phase Cd can condense, adsorb, or react on fly-ash, sorbent, and pre-existing particle surfaces [33,34]. This behavior may favor association with fine or submicrometric particles, although the extent of partitioning depends on Cd speciation, temperature profile, fuel or feedstock composition, chlorine and sulfur chemistry, and the availability and reactivity of particle surfaces [33,34]. Such behavior reflects process-dependent redistribution and should not be extrapolated uniformly to all metals or thermal systems.

Residual-fuel combustion represents a distinct source-related pathway for Ni and vanadium. In an experimental marine-engine study, both metals were detected in soot particles and in bulk PM_2.5_, but the metal burden measured directly within soot was substantially lower than that present in total PM_2.5_ [32]. This finding indicates that Ni and vanadium emitted during heavy-fuel-oil combustion may occur in several particle-associated forms rather than being confined to the refractory carbonaceous soot core.

(iii) Mechanical generation can introduce metal-bearing material into the atmosphere without prior volatilization. Relevant processes include wind erosion of mineral or contaminated soil, disturbance of industrial dust, road-surface abrasion, tyre and brake wear, construction, agricultural activity, and resuspension of previously deposited material [7,8,23,28,29,35,50,51]. Although these processes often contribute strongly to coarse PM, brake wear, road dust, and disturbed surface material may also generate or resuspend particles within PM_2.5_ [8,35,50]. The presence of a metal in fine PM therefore does not, by itself, demonstrate a combustion-related or other high-temperature source.

For Pb, resuspension has particular temporal importance because contaminated urban soils and surface dust may retain material deposited during earlier emission periods [7]. Contemporary atmospheric Pb may consequently represent a combination of ongoing primary emissions and secondary mobilization from legacy reservoirs. Resuspendable fractions smaller than 10 µm may be enriched in potentially toxic elements relative to bulk soil and can contribute to road dust and atmospheric PM_10_ under mechanical disturbance [51]. Cd, Ni, and other metals may likewise be returned to the atmosphere where contaminated urban or industrial surface materials are exposed to erosion or mechanical disturbance [8,51].

(iv) Particle composition may continue to evolve after emission. Condensation of gaseous species onto existing particles, coagulation among initially distinct particle populations, heterogeneous reactions, and association with secondary aerosol components can modify particle size, composition, surface properties, and mixing state during transport [3,6]. The physicochemical matrix surrounding a metal-bearing phase at a receptor site may therefore differ from that of the initially emitted particle.

The origin of the metal-bearing material should consequently be distinguished from the later atmospheric evolution of its carrier particle. A particle collected at a receptor site may contain source-derived metal phases together with constituents acquired during transport and aging. Metal–particle association is therefore not necessarily generated by a single process or preserved unchanged from emission to deposition. These mechanisms generate source-related differences in the distribution of Pb, Cd, and Ni among aerodynamic size fractions. However, size partitioning reflects the combined influence of emission processes, particle formation, atmospheric processing, and subsequent mixing rather than metal identity alone.

The Žilina study illustrates this complexity across particles with aerodynamic diameters of ≤1 µm (PM_1_), PM_2.5_, and PM_2.5–10_ [35]. Cd, Pb, and Zn showed a pronounced presence in PM_1_ and PM_2.5_, particularly during the heating season. By contrast, Cu, Sb, Ba, Ca, Cr, Fe, Mg, and Al were associated more strongly with PM_2.5–10_ [35]. PMF identified tire and brake abrasion, road-dust resuspension and winter salting, and combustion processes as major contributing sources. Combustion contributed more strongly to PM_1_ and PM_2.5_, whereas non-exhaust traffic emissions, road dust, and winter salting were more influential in the coarse fraction [35]. This example illustrates that source-related tendencies may be size-selective without implying that a given metal or source is confined to a single aerodynamic fraction.

### 4.3. Atmospheric Transport, Deposition, and Resuspension

Following emission or resuspension, metal-bearing particles undergo atmospheric dispersion, dilution, aging, and mixing before removal through wet or dry deposition [3,6,9,10,48]. Atmospheric residence time and transport distance depend on aerodynamic size, particle density and morphology, hygroscopicity, emission height, boundary-layer dynamics, atmospheric stability, precipitation, and other meteorological conditions [3,9,10,48]. Many of the same characteristics that influence atmospheric residence also determine removal efficiency, deposition pathway, and spatial distribution.

Particle-size partitioning can substantially influence atmospheric transport and deposition. In the regional emission and atmospheric modeling analysis of Jiang et al. [48], 55% of Pb emissions and 58% of Cd emissions were assigned to the fine mode. Fine-mode particles exhibited greater transport potential, whereas coarse particles settled more readily and showed higher dry-deposition rates. These values are specific to the modeled emission inventory, geographical region, and atmospheric conditions, but they illustrate how particle-size distribution can influence the spatial extent of contamination and the relative importance of local and regional transport.

Wet deposition comprises in-cloud and below-cloud scavenging [9]. In-cloud scavenging occurs when aerosol particles become incorporated into cloud droplets or ice particles and are subsequently removed through precipitation. Below-cloud scavenging occurs when falling rain, snow, or other hydrometeors collect particles during their passage through the atmosphere [9]. Scavenging efficiency is influenced by particle-size distribution, precipitation type and intensity, cloud processing, aerosol composition, hygroscopicity, and metal solubility [9,48].

Wet deposition may transfer both particulate and dissolved or partially solubilized metal fractions to terrestrial and aquatic surfaces. Because elemental solubility in precipitation varies according to metal properties, particle association, and environmental conditions, total wet-deposition flux and dissolved metal flux should be treated as distinct, although related, descriptors [9].

Dry deposition occurs in the absence of precipitation and involves turbulent atmospheric transport toward a receiving surface followed by size-dependent collection processes [10]. Brownian diffusion is particularly relevant to very small particles, whereas interception, inertial impaction, and gravitational settling become increasingly important as particle size increases. Accumulation-mode particles may exhibit comparatively low deposition velocities under certain atmospheric and surface conditions, favoring longer atmospheric residence and regional transport [10]. These mechanisms describe the behavior of the carrier particles and should not be interpreted as intrinsic deposition properties of Pb, Cd, or Ni themselves.

The relative contributions of wet and dry deposition vary among regions, sources, particle-size distributions, and meteorological regimes. In the mainland-China modeling study of Jiang et al. [48], dry-deposition fluxes exceeded wet-deposition fluxes for both Cd and Pb, with modeled dry-to-wet ratios of 2.91 and 1.80, respectively. These values should not be generalized beyond the investigated emission inventory, aerosol-size distribution, meteorology, and precipitation regime.

Deposition transfers metal-bearing particles to soils, vegetation, surface waters, sediments, roads, and built surfaces but does not eliminate the contaminant [9,10,48]. Following deposition, metals may undergo dissolution, sorption–desorption, complexation, precipitation–dissolution, and redistribution among solid, dissolved, and colloidal phases, with subsequent mobility controlled by pH, mineralogy, organic matter, ligand availability, redox conditions, moisture, and hydrological transport [15,16]. The receiving surface further influences subsequent fate: vegetation may intercept and retain particles or promote wash-off, roads and built surfaces may temporarily store deposited material, and soils and sediments may act as persistent reservoirs from which metals can later be redistributed [7,8,9,10,51,52,53].

Atmospheric deposition is consequently not always a permanent endpoint. Metal-bearing material accumulated in soil, road dust, exposed sediment, and other surface reservoirs may return to the atmosphere through wind erosion, vehicle-induced turbulence, road abrasion, construction, agricultural activity, or other mechanical disturbances [7,8,35,51]. Resuspended particles may differ from the originally deposited material because chemical and physical transformations can occur during surface residence [15,16].

For Pb, repeated resuspension is particularly relevant in urban environments containing historically contaminated soils and dust [7]. Contemporary Pb concentrations may therefore reflect both current emissions and remobilization of material accumulated over previous decades. Similar recycling may occur for Cd and Ni where contaminated industrial, roadside, or urban surface reservoirs remain exposed to disturbance [8,51].

Repeated cycles of emission, atmospheric transport, deposition, surface transformation, and resuspension connect present-day PM burdens with both recent sources and persistent environmental reservoirs. Atmospheric concentration at a given time therefore represents a dynamic mixture of newly emitted particles, aged particles, and material remobilized from previously contaminated surfaces.

Representative regional data illustrating PM-associated metal concentrations, particle-size-dependent transport, and atmospheric deposition of Pb, Cd, and Ni are summarized in Table 2. Because concentration and deposition datasets differ in PM fraction, sampling period, site category, analytical design, meteorology, and precipitation regime, the reported values should be interpreted as study- or region-specific observations rather than directly comparable universal coefficients.

### 4.4. Analytical Characterization and Integrated Monitoring of Metal-Containing PM

Characterization of PM-associated Pb, Cd, and Ni should combine particle-size information with elemental composition and, where required by the study objective, information on metal-bearing phases, solubility, or operationally defined mobility. Size-selective sampling of PM_10_, PM_2.5_, or other aerodynamic fractions provides exposure- and source-relevant information that bulk elemental analysis cannot supply, because particle size influences atmospheric transport, respiratory deposition, and the distribution of metals among fine and coarse fractions. However, aerodynamic fraction alone does not establish chemical form, dissolution behavior, pulmonary bioaccessibility, or biological availability [1,11,12,57]. The principal analytical approaches used to characterize PM-associated Pb, Cd, and Ni, together with the information they provide and their key analytical considerations, are summarized in Table 3.

Elemental determination may be performed using techniques such as flame atomic absorption spectrometry (FAAS), graphite furnace atomic absorption spectrometry (GFAAS), inductively coupled plasma optical emission spectrometry (ICP-OES), inductively coupled plasma mass spectrometry (ICP-MS), or X-ray fluorescence (XRF), depending on the matrix, expected concentration range, sample mass, required detection limits, number of target elements, analytical throughput, and study objective. FAAS and GFAAS remain useful for targeted elemental determination, whereas ICP-OES and particularly ICP-MS facilitate multielement analysis over progressively lower concentration ranges; XRF can provide direct, non-destructive elemental characterization of compatible filters or solid matrices. No technique constitutes a universally superior method, and analytical performance must be demonstrated for the specific matrix through appropriate calibration, interference control, recovery assessment, limits of detection and quantification, and reference or quality-control materials [58,59,60,61,62].

Total elemental concentration should be distinguished from operational fractionation, chemical speciation, and bioaccessibility. Sequential-extraction procedures may provide comparative information on operationally defined exchangeable, reducible, oxidizable, or residual fractions, but these fractions are method-defined and are not equivalent to discrete chemical species. Chemical-speciation analysis requires analytical resolution of defined chemical forms or metal-bearing phases, whereas pulmonary bioaccessibility estimates the fraction released from particles under specified physiologically relevant in vitro conditions. Accordingly, total concentration, operational fractionation, chemical speciation, pulmonary bioaccessibility, absorbed dose, and systemic bioavailability represent different analytical or biological levels and should not be used interchangeably [11,12,63,64,65].

Integrated monitoring should therefore follow the hypothesized source-to-exposure pathway rather than apply an identical set of matrices at every site. Depending on the problem investigated, coordinated measurements may involve atmospheric PM, deposited dust, surface soil, vegetation, water or sediment, food-chain matrices, and internal-dose indicators. Measurements obtained from these compartments represent different stages of the source-to-effect continuum and should be linked through compatible sampling designs, analytical methods, concentration bases, reporting units, and quality-assurance procedures.

**Table 3:** Analytical approaches for characterization and monitoring of PM-associated Pb, Cd, and Ni.

Analytical Objective	Representative Approach/ Technique	Information Obtained	Main Strengths/Added Value	Key Analytical Considerations	References
Size-resolved particle collection	PM_10_/PM_2.5_ size-selective samplers; impactors; dichotomous sampling	Metal distribution among operational aerodynamic fractions	Links elemental composition with atmospheric transport, source profiles, and respiratory-deposition relevance	Aerodynamic fraction should be interpreted together with chemical form, solubility, bioaccessibility, sampler characteristics, and collection efficiency.	[1,66]
Targeted elemental determination	FAAS	Concentration of individual metals after appropriate sample preparation	Established and accessible approach for targeted Pb, Cd, or Ni determination at suitable concentrations	Matrix-specific preparation, calibration, background correction, detection limits, and quality-control performance should be demonstrated.	[58]
Trace targeted elemental determination	GFAAS	Trace concentrations of one or a limited number of metals	Higher sensitivity and lower sample-volume requirements than flame atomization for selected applications	Matrix and background effects, furnace program, modifiers, calibration strategy, and recovery should be evaluated for the relevant matrix.	[59]
Multielement elemental analysis	ICP-OES	Simultaneous or sequential concentrations of Pb, Cd, Ni, and supporting elements	Efficient multielement characterization over a broad concentration range	Spectral interference, matrix effects, digestion completeness, calibration, and quality-control materials should be addressed.	[60]
Trace/ultratrace multielement analysis	ICP-MS	Very low elemental concentrations and multielement profiles	Particularly suitable for low-loading PM filters and trace environmental or biological matrices	Contamination control, polyatomic/isobaric interference, matrix effects, drift, internal standards, and method-specific detection limits require explicit quality assurance/quality control.	[61]
Direct solid/filter elemental analysis	XRF	Elemental composition of compatible filters or solid matrices without conventional digestion	Non-destructive and multielement; useful for filter-based PM characterization and rapid screening	Performance depends on substrate, loading, particle-size distribution, homogeneity, calibration, spectral overlap, and element-specific sensitivity.	[62,67]
Operational fractionation	Sequential extraction, including Community Bureau of Reference (BCR)-type procedures	Operationally defined exchangeable/acid-soluble, reducible, oxidizable, and residual fractions	Provides comparative information on potential mobility beyond total concentration	Fractions are method-defined; extraction sequence, redistribution during extraction, matrix type, and protocol comparability should be reported explicitly.	[63,64]
Chemical/ speciation characterization	Species- or phase-resolving analytical approaches	Defined oxidation states, molecular complexes, or metal-bearing phases, depending on the method	Can explain differences in dissolution, mobility, source interpretation, bioaccessibility, and toxicity not captured by total concentration	The selected analytical technique must resolve the chemical form or phase claimed; operational fractionation alone should not be described as true chemical speciation.	[63]
Pulmonary bioaccessibility	Simulated respiratory fluids under defined extracellular or phagolysosomal conditions	Fraction of particle-associated metal released under specified respiratory conditions	Provides an exposure-relevant bridge between total deposited metal and potentially available pulmonary metal	Fluid composition and pH, particle loading, solid-to-liquid ratio, extraction time, agitation, and reporting basis should be standardized or described in sufficient detail for comparison.	[11,12,65]
Analytical quality assurance	Blanks; certified/reference materials; spikes and recoveries; replicates; calibration verification; limits of detection/quantification	Evidence supporting reliability and comparability of measurements	Essential for integration across PM fractions, environmental matrices, sites, and studies	Sampling, preparation, calibration, recovery, contamination control, detection/quantification limits, units, and concentration bases should be harmonized with the analytical objective.	[58,59,60,61,62,67]

Note: Total elemental concentration, operational fractionation, chemical speciation, pulmonary bioaccessibility, absorbed dose, and systemic bioavailability represent distinct analytical or biological levels and should not be interpreted as interchangeable measures.

### 4.5. Atmospheric Dispersion, Deposition, and Source–Receptor Modeling

Atmospheric modeling complements field measurements by estimating the transport, dispersion, deposition, and source–receptor relationships of metal-containing particles across spatial and temporal scales that cannot be resolved by monitoring alone. Model selection should follow the assessment objective because near-source dispersion, long-range transport, regional chemical transport, deposition, and retrospective source attribution represent different modeling problems. For PM-associated Pb, Cd, and Ni, particle-size representation, emission speciation, wet and dry deposition, meteorological inputs, terrain, source configuration, and the availability of observational data for model evaluation are particularly important determinants of model applicability.

The American Meteorological Society/Environmental Protection Agency Regulatory Model (AERMOD) is a steady-state plume model widely used for regulatory near-source dispersion assessment and is suitable when emissions from defined stationary sources are the principal concern [68]. The Hybrid Single-Particle Lagrangian Integrated Trajectory (HYSPLIT) model uses a hybrid Lagrangian–Eulerian framework and is particularly useful for forward or backward trajectories, source–receptor analysis, and transport, dispersion, and deposition over local to global scales [69,70]. The Community Multiscale Air Quality (CMAQ) modeling system is a regional chemical-transport modeling system that integrates emissions, meteorology, atmospheric transformation, transport, and deposition and can explicitly represent particulate toxic metals, including Pb, Cd, and Ni, in fine and coarse particle modes [71,72]. These models are complementary rather than interchangeable because they address different spatial scales, process complexity, and decision contexts.

Model output should not be interpreted as a substitute for measurement. Predictions depend on emission inventories, meteorological fields, particle and deposition parameterization, spatial resolution, boundary conditions, and model structure. Model evaluation should therefore use observations generated with compatible spatial and temporal support, and uncertainty should be reported explicitly. Back trajectories or spatial concentration patterns may support source interpretation but do not independently establish causality, while agreement between modeled and measured concentrations does not demonstrate universal validity outside the evaluated application domain [73].

## 5. Post-Depositional Environmental and Food-Chain Transfer of PM-Associated Pb, Cd, and Ni

Atmospheric deposition provides a pathway through which particulate matter (PM)-associated Pb, Cd, and Ni can enter terrestrial and aquatic food-producing environments. Particle-size partitioning can substantially influence atmospheric transport and deposition. In the regional emission and atmospheric modeling analysis of Jiang et al. [48], 55% of Pb emissions and 58% of Cd emissions were assigned to the fine mode. Fine-mode particles exhibited greater transport potential, whereas coarse particles settled more readily and showed higher dry-deposition rates [48]. These values are specific to the modeled emission inventory, geographical region, and atmospheric conditions, but they illustrate how particle-size distribution can affect the spatial extent of contamination and the relative importance of local and regional transport. Accordingly, the terrestrial and aquatic compartments considered in this section are treated as downstream receiving and transfer compartments for atmospherically deposited PM-associated metals, rather than as independent descriptions of general heavy-metal cycling.

Wet deposition may transfer metal-bearing particles together with dissolved or partially solubilized metal fractions, whereas dry deposition transfers particulate material directly to soils, vegetation, surface waters, and other exposed surfaces [9,10,48,52,53]. Subsequent transfer into food and feed depends on metal speciation, particle characteristics, post-depositional transformation, environmental mobility, biological uptake, and tissue-specific accumulation rather than on atmospheric concentration alone [14,15,16,52,53,74,75].

Food-chain assessment must distinguish atmospheric concentration and deposition flux from post-depositional environmental fractions, external surface contamination, internal biological accumulation, and concentrations measured in the final edible matrix, because these represent related but non-equivalent stages of transfer [14,15,16,52,53,74,75]. Detection of Pb, Cd, or Ni in food does not by itself establish atmospheric PM as the predominant source. Source attribution requires independent evidence because geological background, agricultural inputs, industrial releases, historical contamination, and other environmental pathways may contribute simultaneously [36,37,39,41,42,76].

### 5.1. Atmospheric Deposition to Agricultural Soils and Vegetation

Agricultural soils can act as long-term receptors and chemically reactive storage matrices for atmospherically deposited metals [9,15,16,48]. However, atmospheric deposition represents only one component of the metal balance in cultivated soils, alongside geogenic sources and agricultural inputs or amendments [41,76]. Attribution of measured soil concentrations therefore requires consideration of geological background, historical contamination, land-management practices, and cumulative external inputs [41,76].

Bare soil and vegetated surfaces interact differently with atmospheric particles because they differ in aerodynamic roughness, surface structure, moisture, and particle-interception capacity [9,10,52,53,74]. Crop and forage canopies may intercept metal-bearing particles before they reach the underlying soil, with retention influenced by particle properties, canopy architecture, plant-surface characteristics, and meteorological conditions [10,52,53,74]. After interception, deposited material may remain on aerial surfaces, become incorporated into foliar tissues, be transferred toward the underlying soil by precipitation or irrigation, or return to the atmosphere through resuspension [52,53,74,77]. These atmosphere–plant and atmosphere–soil pathways are therefore interconnected but not equivalent [52,53,74,77].

Once deposited onto soil, PM-associated metals may remain associated with their carrier particles or undergo dissolution, sorption–desorption, ion exchange, complexation, and precipitation, thereby redistributing among relatively retained, mobile, dissolved, and potentially phytoavailable pools [15,16]. The extent of this redistribution depends principally on soil pH, mineralogy, organic matter, competing ions, ligand availability, redox conditions, and hydrology; consequently, similar total soil concentrations may correspond to substantially different fractions available for plant uptake [14,15,16,75].

Post-depositional behavior also differs among Pb, Cd, and Ni. Pb is frequently retained strongly in surface soils through sorption, complexation, and precipitation, although acidification and complexing ligands may increase its mobility; contaminated topsoil and surface dust may subsequently serve as reservoirs for resuspension and redeposition [7,15,16,51]. Cd is generally more mobile than Pb under acidic or low-sorption conditions and may therefore remain comparatively available for crop uptake [14,15,16,75]. Ni mobility is likewise influenced by pH, ligand complexation, mineral surfaces, and organic matter, but elevated soil Ni may also reflect Ni-rich parent materials rather than atmospheric contamination [15,16,41].

Deposited metals may subsequently be redistributed vertically by infiltration or laterally through runoff and erosion, allowing agricultural soils to function not only as receiving and storage compartments but also as secondary sources for transfer toward drainage systems and connected aquatic environments [15,16,78,79].

#### Management of Post-Depositional Metal Reservoirs

Once PM-associated Pb, Cd, and Ni have accumulated in surface soils, remediation may reduce subsequent exposure by removing part of the contaminant inventory, decreasing metal mobility or bioavailability, limiting transfer to vegetation or water, or suppressing erosion and secondary resuspension. Because elemental contaminants cannot be degraded into innocuous products, removal, immobilization, and exposure-pathway interruption represent fundamentally different remediation objectives and should not be evaluated using interchangeable performance criteria. This distinction is particularly important for atmospherically deposited metals because contaminated surface soil can function simultaneously as a long-term reservoir, a source of plant uptake and runoff, and a secondary source of inhalable metal-containing dust [80,81,82,83,84].

Phytoextraction removes metal from the contaminated surface reservoir only when Pb-, Cd-, or Ni-containing biomass is harvested and physically removed from the site, whereas phytostabilization primarily aims to reduce mobility, erosion, runoff, dust generation, and biological transfer while leaving most of the contaminant inventory in place. Microorganism-assisted approaches may modify rhizosphere chemistry, plant tolerance, biomass production, metal uptake, or immobilization and may therefore support either extraction or stabilization depending on the plant–microorganism–soil–metal system. Biochar and related amendments similarly act predominantly through immobilization mechanisms such as sorption, ion exchange, surface complexation, precipitation, and modification of soil physicochemical properties rather than through contaminant removal [80,81,82,83,84,85,86].

Remediation performance should therefore be assessed against the intended exposure-reduction objective rather than by changes in a single analytical endpoint. Increased plant-tissue concentration does not necessarily indicate efficient phytoextraction if biomass production is low, and decreased operationally extractable metal does not demonstrate contaminant removal or reduced pulmonary bioaccessibility. Likewise, enhanced mobilization during chelator-assisted treatment may increase plant uptake but may also increase leaching or off-site transport. Field-scale evaluation should integrate residual soil burden, metal mobility, plant uptake, runoff and leaching, dust-generation or resuspension potential, route-relevant bioaccessibility where applicable, transfer into food or feed, and the persistence of treatment effects across seasons or years [63,64,80,81,82,83,84,87].

The principal remediation strategies for post-depositional soil reservoirs of PM-associated Pb, Cd, and Ni are summarized comparatively in Table 4, with emphasis on their remediation objectives, principal mechanisms, relevant performance indicators, and key considerations for field application.

### 5.2. Soil-to-Plant Transfer and Direct Foliar Contamination

Plants may acquire Pb, Cd, and Ni through two principal and potentially simultaneous pathways: uptake from the rhizosphere through the root system and direct interaction of atmospheric deposition with aerial plant surfaces [14,52,53,74,75]. Concentrations measured in leaves, fruits, grains, or forage may therefore include root-derived internal accumulation, foliar internalization, externally retained particulate material, or combinations of these contributions [14,52,53,74,75]. Neither total atmospheric deposition nor total soil concentration directly predicts the internal concentration of a harvested plant organ; interpretation therefore requires consideration of metal and particle characteristics, soil conditions, plant species and organ, and whether externally deposited material was removed before analysis [14,15,16,52,53,74,75].

#### 5.2.1. Root Uptake, Translocation, and Intracellular Sequestration

Following atmospheric deposition to soil, root uptake depends primarily on the metal fraction accessible at the soil–root interface rather than on total soil concentration, with rhizosphere chemistry and plant-specific transport processes influencing entry and subsequent translocation [14,15,16,75,88]. Metals retained at root surfaces, cell walls, or apoplastic compartments should be distinguished from internally absorbed metal, because only the latter is available for systemic redistribution within the plant [88,89]. After cellular entry, root-to-shoot transport is metal- and species-dependent; for example, xylem-mediated transport contributes importantly to Cd accumulation in rice shoots and grain, whereas Ni uptake and translocation may involve transport systems and ligand interactions that also participate in essential-element homeostasis [90,91,92].

Intracellular binding and sequestration can reduce the mobile cytosolic metal fraction and thereby limit translocation without eliminating the metal from plant tissues [93,94,95]. Pb generally shows stronger root retention and more limited root-to-shoot transfer than Cd in non-hyperaccumulating plants; nevertheless, limited internal translocation does not prevent direct atmospheric contamination of leaves, fruits, grains, or forage [15,16,52,53,74,89,96]. Cd has greater potential for internal transfer through the soil–plant continuum and may reach shoots and edible organs, although accumulation remains strongly species-, cultivar-, tissue-, and soil-dependent [14,75,91,97]. For Ni, uptake and translocation are likewise condition-dependent, and elevated crop concentrations must be interpreted cautiously because Ni-rich parent materials may produce substantial soil–plant exposure independently of atmospheric contamination [41,90,92,98].

#### 5.2.2. Foliar Deposition, Surface Retention, and Uptake

Metal-bearing particles and dissolved or partially solubilized metal fractions may reach leaves, fruits, grains, and forage through dry deposition and precipitation-mediated transfer [9,10,52,53,74]. Initial interception and subsequent retention depend on particle properties, canopy and leaf-surface characteristics, and meteorological conditions [10,52,53,74]. After deposition, material may remain attached to plant surfaces, become embedded in surface microstructures, undergo partial dissolution in surface moisture, be removed by rainfall or irrigation, or return to the atmosphere through resuspension [52,53,74].

Deposition and internalization are not equivalent: foliar uptake requires passage across the plant surface and depends on metal speciation and solubility, particle characteristics, leaf morphology, and contact conditions [52,53,74]. Dissolved metal species may enter through cuticular- or stomatal-associated pathways, whereas intact-particle uptake appears strongly size- and surface-dependent; the relative contribution of these routes remains incompletely resolved [52,53,74]. Following foliar entry, metals may remain locally retained or undergo internal redistribution, but foliar penetration does not necessarily imply efficient transfer to the harvested edible organ [52,53,74].

The analytical distinction between external surface retention and internal uptake is fundamental. Concentrations measured in unwashed aerial plant material may include both externally deposited PM-associated metal and internally accumulated metal, potentially complicating interpretation of the transfer pathway [52,53,74,99,100]. Washing can reduce externally associated Pb in lettuce, although complete removal cannot be assumed because effectiveness depends on contaminant form, particle adhesion, plant-surface properties, and the washing procedure [100].

#### 5.2.3. Plant Accumulation and Food-Chain Relevance

The concentration measured in an edible or feed-relevant plant organ reflects the combined effects of environmental availability, root or foliar entry, internal transport, tissue retention, sequestration, detoxification, and externally retained particulate material [14,52,53,74,75,91,96,97,98,99,100]. Because these contributions are metal-, crop-, organ-, and exposure-dependent, no universal ranking of Pb, Cd, and Ni accumulation is appropriate; representative crop-specific concentrations should instead be interpreted within defined plant organs and analytical conditions, as summarized in Table 5.

Leafy vegetables and forage crops provide extensive surfaces for atmospheric particle interception and retention [52,53,74]. By contrast, concentrations in grains, fruits, roots, and tubers may reflect different combinations of root uptake, vascular transport, direct surface exposure, and post-depositional retention; values measured in leaves should therefore not be extrapolated directly to reproductive or storage organs [52,53,74,91,96,97,98].

Phytotoxicity and food-safety relevance are distinct endpoints. Edible or feed-relevant plant material may contain Pb, Cd, or Ni at concentrations relevant to dietary exposure without visible injury, because metal binding and compartmentation can reduce cellular toxicity without eliminating the metal from the harvested organ; direct chemical analysis of the relevant matrix is therefore required for residue assessment [52,53,74,93,94,95,96,97,98,99,100].

Interpretation of crop-contamination data should specify the crop and harvested organ, atmospheric-exposure context, washing and sample-preparation procedures, and the fresh-, wet-, or dry-weight basis of reported concentrations, because these factors are critical for distinguishing external PM-associated contamination from internal accumulation and for comparing results across studies [14,52,53,74,75,99,100].

### 5.3. Feed-to-Animal Transfer and Residues in Foods of Animal Origin

Feed-to-animal transfer links ingestion of contaminated feed or forage with gastrointestinal release and absorption, systemic distribution, and subsequent residues in edible tissues or animal products. Because these processes are matrix- and species-dependent, feed concentration cannot be translated directly into concentrations in milk, eggs, muscle, liver, or kidney without appropriate toxicokinetic evidence [109,110,111,112,113,114].

#### 5.3.1. Exposure Through Feed, Water, Soil, and Dust

Food-producing animals may be exposed to Pb, Cd, and Ni through contaminated forage or feed, drinking water, and incidental ingestion of soil or dust; in areas influenced by atmospheric deposition, metals may be incorporated into plant tissues, remain externally retained on herbage, or occur in deposited or resuspended soil and dust ingested during grazing [14,52,53,74,75,109,110,111,112,113]. Among food-producing animals, grazing ruminants, particularly cattle and sheep, represent a priority group for soil- and dust-mediated exposure on contaminated pastures. In the field study of Thornton and Abrahams [113], involuntary soil ingestion accounted for approximately 1% to nearly 18% of dry-matter intake in cattle and up to 30% in sheep; on contaminated land, ingested soil contributed approximately 9–80% of total Pb intake in cattle. These values are site- and management-dependent but demonstrate that direct soil ingestion may dominate Pb exposure even when soil-to-plant transfer is limited; externally deposited or resuspended particles on herbage provide an additional PM-related pathway [52,53,74,113]. Consequently, metal concentrations measured in forage or feed do not by themselves identify the contamination pathway or the dose subsequently available for absorption.

Directive 2002/32/EC, as amended, establishes maximum contents for Pb and Cd in specified products intended for animal feed; these values represent feed-compliance thresholds rather than direct predictors of residues in milk, eggs, muscle, liver, or kidney, which depend on species-, tissue-, and exposure-specific transfer kinetics [109,110,114,115].

Total metal concentration in feed does not directly represent absorbed dose: gastrointestinal bioaccessibility depends on chemical form, solubility, particle–matrix association, and dietary conditions, whereas systemic bioavailability additionally requires intestinal absorption [109,110,111,112,116]. Exposure comparisons should therefore distinguish feed concentration from total daily intake and define transfer metrics explicitly, because feed-to-tissue or feed-to-product relationships are species-, matrix-, and exposure-dependent [114].

#### 5.3.2. Metal-Specific Distribution and Retention

Metal-specific distribution determines which tissues are most informative for assessing downstream food-chain transfer. Pb is distributed among blood, soft tissues, and bone, with bone serving as an important long-term retention compartment; concentrations measured in one matrix should therefore not be extrapolated directly to another [20,109,110,114]. Cd preferentially accumulates in liver and kidney, and controlled feeding studies in sheep indicate that both dietary concentration and exposure duration contribute to hepatic and renal accumulation, making cumulative exposure more informative than a single feed measurement for interpreting organ burdens [109,110,117,118]. For Ni, available evidence does not support a fixed relationship between total dietary exposure and concentrations in specific tissues or animal products, and robust feed-to-food carry-over rates remain insufficiently established [111,112,119].

#### 5.3.3. Residues in Milk, Eggs, and Edible Tissues

Milk, eggs, muscle, liver, and kidney represent distinct residue endpoints, and preferential accumulation in one tissue does not imply proportional transfer to other edible tissues or secreted products [109,110,114,117,118,120,121,122]. In lactating cows, controlled dietary exposure resulted in predominant Cd accumulation in liver and kidney without a corresponding dose-related increase in milk or skeletal muscle, whereas Pb produced dose-related increases in blood, milk, bone, liver, and kidney but not a corresponding accumulation pattern in skeletal muscle; bone was the principal long-term Pb deposition site under the experimental conditions [120]. In laying hens, dietary Pb increased concentrations in yolk and albumen as well as in several internal organs, whereas experimental Cd exposure produced hepatic and renal retention, with Cd detected only infrequently in yolk and not in albumen under the conditions examined [121,122].

These examples illustrate that feed-to-food transfer is both metal- and matrix-specific and should not be generalized as a universal carry-over coefficient. Residue studies should therefore specify the animal species, exposure level and duration, analyzed tissue or product, and concentration basis, and transfer parameters derived for one matrix should not be applied directly to another without matrix-specific evidence [109,110,114,117,120,121,122].

### 5.4. Aquatic Transfer of PM-Associated Metals and Contamination of Fishery Products

Aquatic environments may receive PM-associated Pb, Cd, and Ni directly through atmospheric deposition and indirectly after deposition to catchment surfaces followed by runoff or erosion. Other inputs, including wastewater, mining or industrial discharges, and remobilization of contaminated sediments, may contribute simultaneously; consequently, concentrations measured in water, suspended particulate matter, or sediments cannot by themselves quantify the atmospheric contribution [9,48,78,79,123,124].

#### 5.4.1. Partitioning Among Water, Particles, and Sediments

After entering an aquatic system, PM-associated metals may redistribute among dissolved, colloidal, suspended-particulate, pore-water, and sediment-bound fractions, with partitioning controlled principally by metal speciation, pH, redox conditions, salinity, organic matter, and particle characteristics [123,124]. Dissolved and colloidal forms may remain mobile in the water column, whereas particle-associated metals may be transported with suspended matter or deposited into sediments; subsequent changes in water chemistry or hydrodynamic conditions can remobilize these fractions [123,124]. Accordingly, total, dissolved, particulate, and sediment concentrations represent related but non-equivalent indicators and should not be interpreted individually as direct measures of biological availability. Sediments may therefore act as long-term sinks for deposited metal-bearing material but can also become secondary sources when changes in redox or physicochemical conditions, or physical disturbance such as storms, currents, bioturbation, or dredging, remobilize previously retained metals or contaminated particles [123,124,125,126,127].

#### 5.4.2. Metal-Specific Aquatic Mobility

Pb, Cd, and Ni differ in their post-depositional aquatic mobility in ways that can influence biological exposure. Pb is generally strongly particle-reactive and is therefore frequently associated with suspended material and sediments, so low dissolved concentrations do not necessarily indicate a low total aquatic burden [123,124,125,126,127]. Cd is commonly more mobile than Pb, although its partitioning is strongly modified by pH, redox conditions, sorption capacity, and salinity; estuarine salinity gradients may further alter dissolved Cd through complexation processes [123,124,128]. Ni may occur in both dissolved and particle-associated fractions, with dissolved-organic-matter complexation influencing free-ion activity, sorption, transport, and potential biological availability [123,124,129,130]. These differences mean that the relationship between total, dissolved, particulate, and biologically available metal varies among Pb, Cd, and Ni and between freshwater, estuarine, and marine environments.

#### 5.4.3. Aquatic Uptake, Bioaccumulation, and Trophic Transfer

Aquatic organisms may acquire Pb, Cd, and Ni through both aqueous and dietary pathways. Dissolved or bioavailable metal species may be taken up across gills or other permeable surfaces, whereas dietary exposure may occur through ingestion of suspended particles, sediment-associated material, microorganisms, aquatic plants, invertebrates, or fish prey [131,132,133]. The relative importance of these pathways depends on metal speciation and availability together with organism-specific habitat and feeding characteristics; consequently, organisms occupying the same aquatic environment may exhibit substantially different accumulated concentrations [131,132,133].

At the base of pelagic food webs, phytoplankton and zooplankton can act as important transfer compartments for particle-associated and dissolved metals. In a Gulf of Lions food web spanning size-fractionated plankton, sardine, and anchovy, Chouvelon et al. [134] reported strongly size-dependent metal burdens: Ni and Pb generally decreased from plankton toward fish, whereas Cd was highest in intermediate plankton fractions and remained low in fish. Higher trophic consumers, including aquatic mammals, may also contain these metals, but tissue burdens reflect diet, species, age, and metal-specific toxicokinetics rather than trophic position alone. For example, liver samples from 69 seals, porpoises, and dolphins collected around the British Isles contained Cd from <0.06 to 11 µg g^−1^ wet weight and Pb from <0.6 to 4.3 µg g^−1^ wet weight, while Ni was below detection in the analyzed samples [135]. These historical, tissue-specific observations demonstrate occurrence and transfer but should not be interpreted as evidence of universal biomagnification.

Bioaccumulation, trophic transfer, and biomagnification should be distinguished explicitly. Bioaccumulation describes net accumulation from all relevant environmental and dietary sources, whereas trophic transfer refers specifically to transfer from food or prey to a consumer [131,136]. Biomagnification should be concluded only when comparable data demonstrate a systematic increase in concentration from prey to consumer or with increasing trophic position within a defined food web; bioaccumulation in individual organisms does not by itself demonstrate biomagnification [131,136].

#### 5.4.4. Tissue-Specific Accumulation and Edible Aquatic Products

Metal accumulation in fish is strongly tissue- and species-dependent, and concentrations measured in environmental-monitoring organs should not be assumed to represent edible muscle [137,138]. Representative studies include wild marine fish from the South China Sea and the freshwater species *Capoeta umbla* and *Luciobarbus mystaceus* from the Tigris River, illustrating that Pb, Cd, and Ni assessment spans markedly different marine and freshwater exposure settings [137,138]. In fish, gills are directly exposed to water and suspended material, whereas liver and kidney contribute to internal uptake, retention, and elimination; skeletal muscle frequently contains lower concentrations of several metals than these metabolically active organs, although the magnitude and direction of tissue differences remain metal-, species-, and environment-dependent [137,138].

Bivalve molluscs and crustaceans provide additional pathways relevant to seafood exposure. Mussels integrate dissolved, particulate, and dietary metal uptake through filtration and ingestion of suspended material, and Pb, Cd, and Ni concentrations may differ among gills, digestive gland, gonad, adductor muscle, and whole soft tissue [131,132,133,139]. For example, Abderrahmani et al. [139] evaluated seasonal Pb, Cd, and Ni distribution in several mussel tissues from the Algerian coast. Crustaceans likewise show organ-specific accumulation; in crabs from coastal waters of southeastern India, metal distribution was evaluated across organs in relation to human consumption risk, emphasizing that hepatopancreatic or cephalothoracic concentrations should not be assumed to represent edible muscle [140].

For food-safety interpretation, environmental-monitoring tissues and edible matrices must be distinguished explicitly. Gills, liver, kidney, digestive gland, and hepatopancreas may provide sensitive indicators of environmental exposure but do not necessarily represent the concentrations consumed by humans; for filleted fish, the relevant matrix is generally muscle, whereas whole-organism or whole-soft-tissue measurements may be appropriate for commodities consumed whole [137,138,139,140,141]. Comparisons with regulatory maximum levels must therefore use the anatomical portion and commodity definition specified by the applicable regulation rather than concentrations measured in water, sediment, whole organisms, or non-edible accumulation organs [141]. 

Representative concentrations illustrating marine and freshwater occurrence and tissue-specific accumulation are summarized in Table 6. The reported values span different tissues and concentration bases and are therefore intended as representative examples rather than as a universal ranking of species or aquatic systems.

### 5.5. Food Safety, Dietary Exposure, and Regulatory Interpretation

Food-safety interpretation of downstream PM-associated metal transfer requires distinction among the concentration measured in a defined edible matrix, the legally applicable maximum level, estimated dietary exposure, and the toxicological reference framework used for risk characterization. These quantities represent different endpoints and should not be treated as interchangeable [141,148,149,150,151,152].

Within the EU, Regulation (EU) 2023/915, as amended, establishes commodity-specific maximum levels for Pb and Cd and, following Regulation (EU) 2024/1987, for Ni in specified food categories [141,148]. Regulatory comparison is valid only when the analyzed matrix, edible portion, preparation state, and concentration basis correspond to the applicable regulatory entry. This distinction is particularly important for PM-related contamination because environmental studies may analyze unwashed plants, whole aquatic organisms, sediments, or non-edible accumulation organs that are not directly comparable with regulatory food matrices [52,53,74,99,100,137,138,139,140,141,148].

The regulatory framework is metal- and commodity-specific. For example, Regulation (EU) 2023/915 establishes general maximum levels of 0.10 mg/kg wet weight for Pb in meat from specified food-producing species, 0.020 mg/kg for Pb in raw and heat-treated milk, and 0.30 mg/kg wet weight for Pb in fish muscle; for Cd, general maximum levels of 0.050 mg/kg wet weight apply to meat from specified species and to fish muscle, with different or higher levels applying to defined offal and aquatic-food categories [141]. By contrast, the Ni limits introduced by Regulation (EU) 2024/1987 apply predominantly to specified plant-derived and other selected food categories rather than providing a parallel set of maximum levels for meat, milk, fish, crustaceans, or molluscs. The absence of a commodity-specific Ni maximum level should not be interpreted as evidence of absent contamination or dietary exposure [141,148,151].

Legal compliance should be distinguished from dietary exposure (DE), which integrates contaminant concentration with food consumption and body weight (BW). For a defined food category, dietary exposure can be estimated from contaminant concentration (C), ingestion rate (IR), and BW as

(1) $$DE=\frac{C\times IR}{BW}$$

where C is the contaminant concentration in the food and IR is the ingestion rate of that food [149,150,151,152]. Aggregate dietary exposure requires summation across relevant food categories and consideration of consumption patterns, body weight, and susceptible or high-consumption groups; consequently, a frequently consumed food with a moderate contaminant concentration may contribute more to chronic intake than a more highly contaminated food consumed infrequently.

Risk characterization should use metal-specific reference frameworks rather than treating dietary-exposure estimates as directly comparable across Pb, Cd, and Ni. For Cd, EFSA established a tolerable weekly intake (TWI) of 2.5 µg/kg BW per week; for Pb, EFSA concluded that the previous provisional tolerable weekly intake was no longer appropriate because no clear threshold could be identified for the critical adverse effects; consequently, a new health-based guidance value was not established, and risk characterization was based on endpoint-specific benchmark-dose-derived reference points and margins of exposure; for Ni, EFSA established a chronic tolerable daily intake (TDI) of 13 µg/kg BW per day and applies a separate margin-of-exposure approach to acute dietary exposure in Ni-sensitized individuals [149,150,151]. These metrics address different toxicological endpoints and averaging periods and should therefore not be interpreted interchangeably.

Regulatory compliance, dietary exposure, toxicological risk, and source attribution represent distinct levels of interpretation. A food may comply with a legal maximum level while still contributing to aggregate dietary exposure, whereas exceedance of a maximum level does not by itself quantify toxicological risk. Likewise, detection of Pb, Cd, or Ni in an edible matrix does not establish atmospheric PM as the predominant source. Attribution of food-chain contamination to atmospheric deposition requires supporting spatial, temporal, chemical, isotopic, or source-apportionment evidence linking atmospheric emissions and deposition with downstream environmental and biological compartments [36,37,39,41,42,141,148,149,150,151]. Atmospheric deposition should therefore be regarded as a potentially important pathway within a multi-source environmental system rather than assumed to be the dominant source whenever these metals are detected in food.

### 5.6. Predictive Modeling of Environmental Transfer and Exposure

Empirical and spatial models can support interpretation of post-depositional transfer by relating measured metal concentrations across environmental matrices and by identifying spatial patterns that warrant additional investigation. Soil-to-plant transfer factors provide a simple concentration-ratio index, whereas multivariable regression can incorporate covariates such as pH, organic matter, extractability, crop characteristics, and irrigation conditions. Geostatistical approaches, including kriging, extend measured data spatially to estimate concentrations at unsampled locations and to delineate gradients or hotspots. These approaches are most informative when the concentration basis, sampling design, spatial scale, and environmental conditions represented by the underlying data are stated explicitly [153,154,155,156].

Exposure models may be deterministic or probabilistic depending on the assessment objective. Deterministic calculations use fixed values for inputs such as contaminant concentration, inhalation or ingestion rate, exposure frequency and duration, body weight, and bioaccessibility, whereas probabilistic approaches represent selected inputs as distributions and propagate their variation through the model, commonly by Monte Carlo simulation. Sensitivity analysis can then identify the parameters or assumptions that contribute most strongly to the modeled outcome. Variability among individuals, locations, or behaviors should be distinguished from uncertainty arising from incomplete knowledge of parameters, model structure, or exposure pathways [157,158].

Data-driven methods, including machine-learning models, can represent nonlinear relationships among environmental, particle, biological, and intervention variables when the prediction target and applicability domain are clearly defined. For the complete PM-associated metal pathway, this review therefore favors a modular framework rather than forcing atmospheric transport, post-depositional reservoirs, environmental transfer, exposure, and intervention into a single model, because these processes operate at different spatial and temporal scales. Reliable integration requires compatibility of units, concentration bases, particle or chemical fractions, spatial and temporal resolution, and uncertainty characterization across linked modules; model outputs should be evaluated against measurements appropriate to the process being represented [73,159,160,161,162,163].

The principal modeling approaches considered across the source-to-exposure pathway are summarized comparatively in Table 7.

## 6. Health Effects of Exposure to PM-Associated Pb, Cd, and Ni

PM-associated Pb, Cd, and Ni become toxicologically relevant primarily when inhaled particles deposit within the respiratory tract and release metal species at pulmonary surfaces or are retained sufficiently long to permit local or systemic effects. Following dissolution and absorption across respiratory barriers, metals may enter the circulation and reach distant tissues and organs. A secondary gastrointestinal contribution may arise when particles cleared from ciliated respiratory regions are swallowed, whereas direct dietary and dermal exposures are considered here only where necessary to distinguish them from the inhalation-derived PM pathway [11,12,20,21,23,119].

The biological effects of PM-associated metals depend not only on external concentration but also on inhaled or ingested dose, regional respiratory deposition, physicochemical and mineralogical form, particle dissolution, bioaccessibility, absorption efficiency, tissue distribution, intracellular retention, and host susceptibility [11,12,13,20,21,23,57,119]. Consequently, equal total concentrations of Pb, Cd, or Ni in air, dust, water, or food may produce substantially different internal and biologically effective doses [11,12,13,20,21,23,119].

Although Pb, Cd, and Ni may converge on oxidative imbalance, mitochondrial dysfunction, inflammatory signaling, DNA injury, altered genome maintenance, epigenetic remodeling, and chromosome-level abnormalities, their initiating mechanisms and toxicokinetic behavior differ substantially [20,21,22,23,118,119]. Pb is characterized by extensive erythrocyte association and long-term skeletal storage; Cd by metallothionein-associated hepatic and renal retention; and Ni by chemical-form-dependent absorption, systemic elimination, and, for poorly soluble particles, potentially prolonged pulmonary persistence [20,21,23,118,119].

### 6.1. From Inhaled PM to Internal Dose: Respiratory Deposition, Pulmonary Retention, and Metal Bioaccessibility

For PM-associated metals, atmospheric concentration, inhaled mass, respiratory deposited dose, bioaccessible metal, absorbed dose, internal dose, and biologically effective dose represent related but non-equivalent stages of exposure. Only a fraction of inhaled particles deposits within the respiratory tract, and only a fraction of the metal associated with deposited particles may be released and cross respiratory barriers; conversely, poorly soluble particles may contribute to a persistent local pulmonary dose even when systemic transfer is limited. Bioaccessibility describes the fraction released from a particle or matrix under defined physiologically relevant conditions, whereas bioavailability requires actual absorption across a biological barrier. Accordingly, total airborne or particle-associated Pb, Cd, or Ni concentration should not be interpreted directly as absorbed dose or target-organ dose [11,12,20,21,23,57,119].

For inhaled PM-associated metals, ambient concentration and inhaled mass are not equivalent to deposited respiratory dose or absorbed dose. Regional deposition depends on aerodynamic diameter, particle density and shape, breathing pattern, ventilation rate, airway geometry, and aerosol physicochemical properties. Larger particles are preferentially deposited in extrathoracic and conducting-airway regions through mechanisms including inertial impaction and gravitational settling, whereas smaller particles have a greater probability of penetrating into bronchiolar and alveolar regions. However, PM_10_ and PM_2.5_ are operational aerodynamic size fractions rather than anatomical deposition boundaries, and deposition occurs across a continuous size spectrum; therefore, neither all PM_10_ should be assumed to remain in the upper respiratory tract nor all PM_2.5_ to reach the alveolar region [1,57].

Following deposition, particle fate depends on both the anatomical site of deposition and physicochemical properties. Material deposited in ciliated airways may undergo mucociliary transport toward the pharynx and subsequently be swallowed, providing a secondary gastrointestinal pathway derived from an initially inhaled dose. Particles reaching deeper pulmonary regions may remain locally retained, dissolve in respiratory lining fluid, undergo cellular uptake, or release soluble metal species capable of crossing the respiratory epithelium. Consequently, persistence of the particulate carrier, dissolution of the metal-bearing phase, pulmonary bioaccessibility, and systemic transfer represent distinct processes that should not be treated as equivalent [11,12,20,21,23,57,119].

Metal-specific behavior further modifies the relationship between deposition and internal dose. For Pb-bearing aerosols, respiratory clearance and absorption depend strongly on particle size, chemical form, and solubility; submicrometric Pb-containing particles may reach bronchiolar and alveolar regions, whereas larger particles deposited in ciliated airways are more likely to undergo mucociliary clearance and subsequent swallowing [20]. For Cd, the Nordberg–Kjellström toxicokinetic model illustrates the effect of particle size: particles with a mass median aerodynamic diameter (MMAD) of 5 µm were assigned approximately 75% nasopharyngeal, 20% alveolar, and 5% tracheobronchial deposition, whereas particles with an MMAD of 0.05 µm were assigned approximately 50% alveolar and 10% tracheobronchial deposition, with the remainder largely considered exhaled. These values are model-specific assumptions rather than universal deposition coefficients, but they demonstrate how particle size can alter pulmonary retention and subsequent Cd absorption [21,118]. For Ni-containing aerosols, aerodynamic size interacts strongly with compound solubility: soluble Ni compounds may release Ni^2+^ relatively rapidly, whereas poorly soluble particulate phases may persist at pulmonary deposition sites and provide a prolonged local source through gradual dissolution [23,119].

Pulmonary bioaccessibility is therefore not a fixed proportion of total particle-associated metal concentration. It depends on the metal-bearing phase, particle size and accessible surface area, dissolution kinetics, composition and pH of the simulated respiratory fluid, extraction time, solid-to-liquid ratio, and experimental protocol. A poorly soluble particle may maintain a substantial local pulmonary burden despite limited systemic transfer, whereas partial dissolution may release ions or soluble complexes capable of interacting with epithelial surfaces or entering the circulation. Accordingly, total metal concentration and experimentally measured in vitro pulmonary bioaccessibility should be reported as complementary rather than interchangeable exposure descriptors [11,12,20,21,23,119].

Particles deposited in ciliated respiratory regions may undergo mucociliary clearance and subsequently be swallowed, creating a secondary gastrointestinal component of an initially inhaled PM dose. The fraction of metal released from these particles in the gastrointestinal tract depends on chemical form, mineral phase, particle characteristics, gastrointestinal conditions, and the surrounding matrix, whereas systemic exposure additionally requires absorption across the intestinal epithelium. Oral absorption differs substantially among Pb, Cd, and Ni and is influenced by host- and matrix-related factors; however, because the present review focuses on PM-associated exposure, detailed dietary and gastrointestinal toxicokinetics are not considered further here. The key implication for inhaled PM is that mucociliary clearance can redistribute part of the deposited particulate burden from the respiratory tract to the gastrointestinal tract, so respiratory deposition and subsequent swallowing should be interpreted as sequential components of the same initial inhalation exposure rather than as independent exposure events [13,20,21,23,57,119].

Dermal absorption is generally limited for inorganic Pb and Cd relative to inhalation and ingestion, whereas penetration of PM-associated metals through skin can increase when barrier integrity is compromised. For Ni, local cutaneous exposure is additionally important because of its well-established sensitizing potential [23,119,165].

### 6.2. Toxicokinetics, Target-Organ Distribution, and Systemic Toxicity

Following pulmonary absorption of bioaccessible metal released from inhaled particles, Pb, Cd, and Ni exhibit increasingly metal-specific toxicokinetic behavior. Once in the circulation, they differ in chemical form, protein binding, tissue distribution, storage, redistribution, and elimination, producing markedly different relationships among recent exposure, circulating concentration, cumulative body burden, and target-organ dose. Pb is characterized by extensive erythrocyte association and long-term skeletal storage; Cd by metallothionein-associated hepatic and renal retention; and Ni by chemical-form-dependent distribution and comparatively more rapid systemic elimination, although poorly soluble Ni-containing particles may persist locally in the respiratory tract [20,21,23,118,119].

#### 6.2.1. Pb: Blood Distribution, Skeletal Storage, and Target-Organ Toxicity

Following systemic absorption, Pb is distributed among blood, soft tissues, and mineralized tissues. Most circulating Pb is associated with erythrocytes, whereas generally less than 1% of total blood Pb is present in plasma; despite its small proportion, the plasma fraction represents a more readily exchangeable pool available for tissue distribution. Blood Pb is therefore useful as a biomarker of recent or ongoing absorbed exposure but does not fully represent cumulative body burden because circulating Pb also reflects exchange with endogenous tissue stores, particularly bone [20].

Pb initially distributes to highly perfused tissues, including the liver, kidneys, and brain, but mineralized tissues constitute the principal long-term storage compartment. Estimates summarized by ATSDR indicate that approximately 94% of total Pb body burden in adults and 76% in children may reside in bone, although these are population estimates rather than fixed individual values. Skeletal Pb can persist for years to decades and remains partly exchangeable with circulating pools. Consequently, increased bone resorption during physiological or pathological states can remobilize previously stored Pb, allowing internal exposure to persist even after external exposure has declined [20].

Major systemic targets of Pb include the nervous, hematopoietic, cardiovascular, and renal systems. The developing nervous system is particularly sensitive, with Pb exposure associated with cognitive, behavioral, neuromotor, and neurosensory impairment; mechanistically, Pb can disturb Ca^2+^-dependent signaling, ion-channel function, neurotransmitter release, and synaptic plasticity. Hematological toxicity includes inhibition of heme biosynthesis, particularly through effects on δ-aminolevulinic acid dehydratase and ferrochelatase, thereby reducing efficient hemoglobin synthesis. Cardiovascular effects include increased blood pressure and hypertension. Prolonged exposure may also affect renal tubular and glomerular function, and renal target-organ dose may reflect both current circulating Pb and endogenous release from cumulative skeletal stores [19,20].

#### 6.2.2. Cd: Metallothionein-Associated Retention, Renal Accumulation, and Systemic Toxicity

Following systemic absorption, Cd is transported in blood in association with proteins and other ligands and accumulates predominantly in the liver and kidneys. Its exceptionally slow elimination reflects strong intracellular binding, particularly to metallothionein (MT), and limited daily excretion. Toxicokinetic models summarized by ATSDR estimate apparent biological half-times of approximately 4–19 years in the human liver and 6–38 years in the kidney; these ranges are model-derived rather than universal constants but illustrate the pronounced cumulative persistence of Cd [21,118].

After hepatic uptake, Cd induces MT synthesis and becomes sequestered in Cd–MT complexes and other intracellular pools. This binding reduces the immediately reactive Cd^2+^ fraction but simultaneously favors prolonged retention. Cd–MT released into the circulation can undergo glomerular filtration and proximal-tubular reabsorption; lysosomal degradation of the protein component then releases Cd within tubular cells, where renewed MT binding promotes renal cortical accumulation. Metallothionein therefore acts as both a protective intracellular buffer and a determinant of long-term Cd persistence and redistribution rather than as a purely detoxifying mechanism [21,118].

The proximal tubule is a principal systemic target of chronic Cd toxicity, and progressive renal cortical accumulation may impair tubular reabsorption and promote low-molecular-weight proteinuria and other indicators of proximal tubular injury. Chronic renal dysfunction can additionally disturb Ca and phosphate homeostasis and contribute to impaired bone mineralization, osteoporosis, osteomalacia, and increased fracture susceptibility. For inhaled PM-associated Cd, the respiratory tract is simultaneously a portal of systemic uptake and a local target: deposited Cd-containing particles may cause epithelial injury, inflammation, impaired pulmonary function, and structural lung damage, whereas absorbed Cd can subsequently accumulate over years in the kidney. Local pulmonary toxicity and chronic systemic renal toxicity should therefore be regarded as distinct but potentially concurrent outcomes of inhaled Cd exposure [21,118].

#### 6.2.3. Ni: Solubility-Dependent Distribution, Pulmonary Persistence, and Systemic Elimination

The toxicokinetics of Ni are strongly influenced by chemical form, compound solubility, and particle characteristics. Following systemic absorption, Ni is distributed to several tissues and is eliminated predominantly through the kidneys. In contrast to Pb and Cd, available evidence does not indicate long-term skeletal storage comparable to Pb or prolonged hepatic–renal retention comparable to Cd. For inhaled poorly soluble Ni-containing particles, however, relatively rapid systemic elimination of absorbed Ni does not imply equally rapid clearance of the pulmonary particulate burden. Local retention, intracellular uptake, phagocytic clearance, and gradual dissolution may maintain a continuing source of Ni^2+^ within respiratory tissues; pulmonary persistence and systemic elimination should therefore be regarded as kinetically distinct processes [23,119].

The respiratory tract is the principal target of repeated inhalation exposure to Ni and Ni compounds. Reported effects include upper- and lower-airway irritation and inflammation, chronic bronchitic changes, impaired pulmonary function, occupational asthma, and, under prolonged high-exposure conditions, structural respiratory injury. The nature and severity of these effects depend on Ni species, solubility, particle size, exposure concentration and duration, pulmonary clearance, and co-exposure conditions. Tissue detection alone should not be interpreted as evidence that a given organ is a principal toxicological target, because chemical form, subcellular localization, residence time, and cellular susceptibility also determine toxicity [23,119].

Interpretation of Ni toxicity therefore requires strict distinction among chemical forms and exposure settings. Nickel carbonyl is a volatile molecular compound with toxicokinetic behavior distinct from conventional particle-associated atmospheric Ni and can produce severe acute pulmonary and systemic toxicity following inhalation; its effects should not be extrapolated directly to metallic Ni, Ni oxides, Ni sulfides, soluble Ni salts, or other PM-associated particulate phases. Likewise, findings obtained under occupational high-exposure conditions or with highly soluble compounds should not be transferred uncritically to low-dose environmental exposure involving poorly soluble Ni-containing PM [23,119].

**Figure 1:**
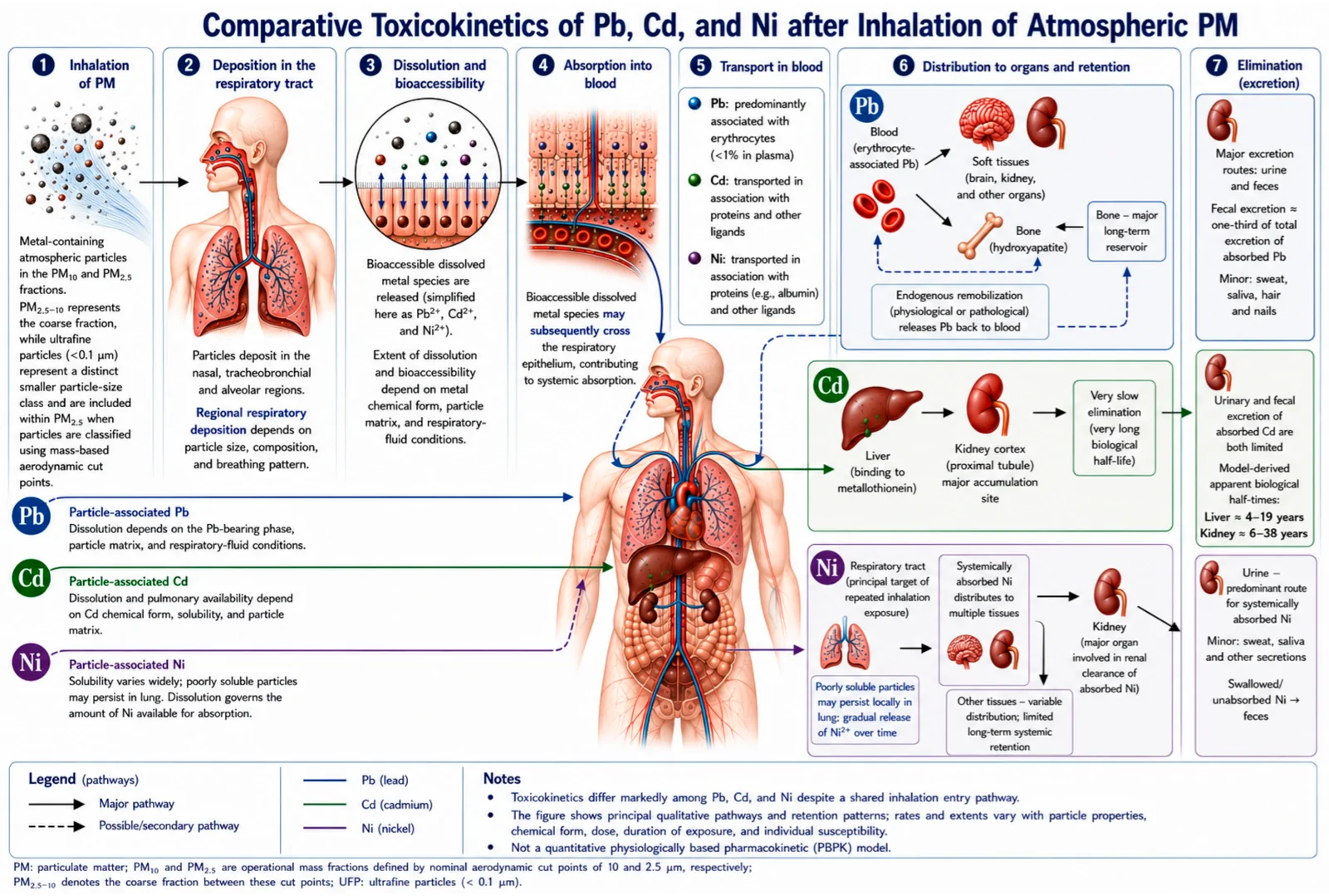
Comparative toxicokinetic redistribution and persistence of inhaled PM-associated Pb, Cd, and Ni in the human body. Following respiratory deposition, dissolution of bioaccessible metal species permits pulmonary absorption and systemic distribution, but subsequent toxicokinetic behavior differs markedly among the three metals. Pb is transported predominantly in association with erythrocytes and progressively accumulates in mineralized tissues, which constitute the principal long-term reservoir and can subsequently remobilize Pb into the circulation. Cd is retained predominantly in the liver and kidneys, with metallothionein-mediated binding contributing both to intracellular sequestration and to prolonged hepatic–renal persistence and redistribution. In contrast, systemically absorbed Ni is eliminated comparatively rapidly, predominantly through the kidneys, whereas poorly soluble Ni-containing particles may remain locally retained in respiratory tissues and progressively release Ni^2+^. Numerical values shown are representative toxicokinetic estimates and should not be interpreted as fixed individual coefficients or as a quantitative mass-balance model [20,21,23,118,119].

The contrasting post-absorption distribution, tissue retention, redistribution, and elimination patterns of Pb, Cd, and Ni following inhalation of metal-containing PM are summarized comparatively in Figure 1.

### 6.3. Oxidative Stress, Inflammation, Mitochondrial Dysfunction, and Cellular Adaptation

Oxidative imbalance, mitochondrial dysfunction, inflammatory signaling, and cellular adaptation form an interconnected response network rather than independent toxicological pathways. Pb and Cd act predominantly as indirect pro-oxidants through disruption of thiol homeostasis, antioxidant defenses, essential-metal regulation, Ca^2+^-dependent signaling, and mitochondrial function, whereas Ni-associated responses depend particularly on chemical form, Ni^2+^ availability, cellular uptake, intracellular dissolution, and the persistence of poorly soluble particles. The magnitude and direction of these responses are additionally influenced by exposure concentration and duration, intracellular metal availability, target-cell phenotype, and the composition of the surrounding PM mixture. Consequently, changes in reactive oxygen species (ROS) production, antioxidant markers, mitochondrial function, or inflammatory mediators should be interpreted as complementary and context-dependent endpoints rather than interchangeable evidence of a single common mechanism [20,21,22,23,24,25,118,119,166].

#### 6.3.1. Oxidative Stress and Mitochondrial Dysfunction

Pb and Cd promote oxidative imbalance predominantly through indirect mechanisms rather than direct catalytic redox cycling. Pb interferes with heme biosynthesis, thiol-dependent defenses, Ca^2+^ signaling, and mitochondrial function, whereas Cd interacts strongly with sulfhydryl-containing molecules, disrupts glutathione and protein-thiol homeostasis, modifies antioxidant-enzyme activity, perturbs essential-metal regulation, and impairs mitochondrial function. These disturbances can increase lipid and protein oxidation, alter redox-sensitive signaling, and amplify secondary ROS generation [20,21,118,166].

For Ni, oxidative and mitochondrial effects depend strongly on chemical form, solubility, cellular uptake, and intracellular Ni^2+^ availability. Soluble compounds may provide a relatively rapid source of biologically available Ni^2+^, whereas poorly soluble particles may persist within respiratory tissues or intracellular compartments and release Ni progressively. Ni may consequently disturb metal-dependent proteins, antioxidant balance, mitochondrial membrane potential, redox-sensitive signaling, and apoptotic pathways, but findings obtained with one Ni form should not be generalized automatically to other particulate or soluble forms [22,23,119].

Glutathione and metallothionein represent complementary but non-equivalent components of cellular defense. The reduced glutathione (GSH)/oxidized glutathione disulfide (GSSG) system provides a dynamic redox buffer involved in peroxide metabolism, electrophile detoxification, and maintenance of protein-thiol status, whereas metallothioneins provide cysteine-rich metal-binding capacity that can reduce the immediately reactive intracellular metal fraction. Changes in total GSH, GSSG, the GSH/GSSG ratio, glutathione-dependent enzyme activity, or metallothionein expression therefore reflect different aspects of cellular response and should not be treated as interchangeable measures of oxidative stress or successful adaptation [118,166].

Mitochondria are both targets and amplifiers of metal-associated oxidative injury. Disturbance of electron transfer, membrane potential, Ca^2+^ handling, redox coupling, or oxidative phosphorylation can reduce adenosine triphosphate production and increase secondary ROS formation; the specific pattern and persistence of mitochondrial dysfunction vary among Pb, Cd, and Ni according to chemical form, intracellular availability, dose, and cell type. Within ambient PM, however, these mechanisms operate in a complex mixture containing other metals, redox-active organic compounds, carbonaceous material, inorganic ions, and reactive particle surfaces. Accordingly, the oxidative potential of the complete aerosol cannot be inferred directly from total Pb, Cd, Ni, or aggregate metal concentration, and statistical association between a metal and PM oxidative potential should not be interpreted automatically as component-specific causality [20,21,22,23,24,25,119].

#### 6.3.2. Inflammatory and Immune Signaling

Oxidative imbalance, mitochondrial dysfunction, and cellular injury can activate inflammatory signaling, while activated immune cells may generate additional ROS and reactive nitrogen species, thereby reinforcing local tissue injury during repeated or persistent exposure. The magnitude and persistence of this response depend on metal identity and chemical form, particle characteristics, intracellular dose, target tissue, exposure duration, and clearance efficiency. Pb-, Cd-, and Ni-associated inflammation should therefore be interpreted as part of an interconnected stress-response network rather than as independent downstream endpoints [20,21,22,23,118,119].

Metal-specific inflammatory patterns nevertheless differ. Pb exposure has been associated with altered redox-sensitive signaling, cytokine regulation, and immune-cell activation, whereas Cd-associated inflammation occurs in the context of prolonged intracellular retention, mitochondrial injury, and organ-specific accumulation, particularly in the respiratory and renal systems. For Ni, inflammatory responses are strongly dependent on chemical form and exposure context; poorly soluble Ni-containing particles may sustain local pulmonary inflammation through prolonged retention and gradual dissolution, whereas Ni^2+^ can also activate immune pathways relevant to sensitization. The latter mechanism, including direct activation of human Toll-like receptor 4 (TLR4), is well established for Ni hypersensitivity but should not be generalized as the dominant pathway for all respiratory or PM-associated Ni exposures [20,21,22,23,118,119,167].

Inflammasome signaling, particularly the NOD-, leucine-rich repeat- and pyrin domain-containing protein 3 (NLRP3) inflammasome, provides a plausible mechanistic interface among particle uptake, lysosomal or mitochondrial injury, ROS formation, and inflammatory amplification. However, much of the mechanistic evidence derives from particles such as silica or asbestos or from experimental manipulation of mitochondrial quality control rather than from defined Pb-, Cd-, or Ni-containing PM. NLRP3 involvement in PM-associated metal toxicity should therefore be regarded as context-dependent and should be inferred only where direct evidence supports inflammasome assembly or caspase-1 activation; ROS formation or mitochondrial injury alone is insufficient to establish NLRP3-mediated toxicity [168,169].

#### 6.3.3. Cellular Adaptive Responses: Antioxidant, Metal-Responsive, and Metallothionein Pathways

Cells exposed to metal-associated oxidative or electrophilic stress may activate cytoprotective transcriptional programs, among which the Kelch-like ECH-associated protein 1 (KEAP1)–nuclear factor erythroid 2-related factor 2 (NRF2) pathway is particularly important. Under basal conditions, KEAP1 promotes NRF2 turnover through a Cullin 3 (CUL3)-dependent ubiquitination system, whereas oxidative or electrophilic modification of this regulatory process permits NRF2 stabilization, nuclear accumulation, and activation of antioxidant-response-element-dependent transcription. NRF2-responsive genes support glutathione synthesis and metabolism, electrophile conjugation, antioxidant defense, and other protective functions. However, increased NRF2 abundance or induction of individual NRF2-responsive genes demonstrates pathway engagement rather than successful restoration of cellular homeostasis [170,171,172].

Metal-responsive adaptation is also mediated through the metal-responsive transcription factor 1 (MTF-1)–metal response element (MRE) system, which regulates metallothionein expression and other components of metal homeostasis. Metallothioneins are cysteine-rich proteins that buffer intracellular metals and can reduce the immediately reactive metal fraction. Their toxicological role is especially important for Cd, for which metallothionein binding provides short-term sequestration but also contributes to prolonged tissue retention, redistribution, renal delivery, and renal accumulation. Metallothionein induction should therefore not be interpreted as an exclusively protective response, and the Cd–metallothionein pathway should not be assumed to operate identically for Pb or Ni because the three metals differ substantially in ligand affinity, intracellular distribution, persistence, and elimination [21,118,166,173].

The biological significance of these adaptive responses depends on exposure intensity and duration. During limited or transient exposure, NRF2 activation, restoration of glutathione-dependent defenses, metallothionein induction, sequestration of labile metal ions, and mitochondrial recovery may contribute to re-establishment of cellular homeostasis. During persistent PM-associated exposure, however, continued metal uptake or intracellular release may coexist with antioxidant and metal-binding responses while oxidative imbalance, mitochondrial dysfunction, inflammatory signaling, and intracellular metal retention persist. Adaptive-marker induction should therefore be interpreted together with functional endpoints such as redox status, mitochondrial function, inflammatory mediators, intracellular metal burden, and cell viability rather than as evidence that toxicity has been successfully prevented [20,21,22,23,118,119,166,170,171,172,173].

### 6.4. DNA Damage, Replication Stress, and Repair Impairment

DNA injury associated with PM-related Pb, Cd, and Ni encompasses multiple, non-equivalent endpoints, including oxidatively modified bases, apurinic/apyrimidinic sites, single- and double-strand breaks, replication-associated lesions, and interference with DNA-damage recognition or repair. These effects arise predominantly through indirect mechanisms involving oxidative imbalance, mitochondrial dysfunction, inflammatory signaling, disruption of thiol- or metal-dependent proteins, and inhibition of selected genome-maintenance processes. Their relative importance differs among Pb, Cd, and Ni according to chemical form, intracellular availability, particle behavior, persistence, and molecular target; consequently, evidence obtained with soluble compounds or high experimental doses should not be extrapolated quantitatively to environmental PM-associated exposure [20,21,22,23,118,119,166,174,175].

#### 6.4.1. DNA Damage and Replication Stress

Oxidative DNA damage provides a mechanistic link between metal-associated redox disturbance and genome instability. Reactive species can modify nucleobases or the deoxyribose–phosphate backbone, producing lesions such as 8-oxo-7,8-dihydroguanine (8-oxoG), apurinic/apyrimidinic (AP) sites, single-strand breaks (SSBs), and, under appropriate conditions, double-strand breaks (DSBs). These endpoints are biologically distinct: for example, unrepaired 8-oxoG can promote G:C→T:A transversions during replication, whereas strand breaks may arise directly or during processing of damaged bases. Accordingly, detection of one lesion type should not be interpreted as a quantitative surrogate for total DNA damage [176,177,178].

Persistent DNA lesions become particularly consequential when they interfere with replication. Replication-fork slowing or uncoupling can generate replication protein A (RPA)-coated single-stranded DNA and activate ataxia telangiectasia and Rad3-related (ATR)-dependent replication-stress signaling, whereas unresolved or inaccurately processed lesions may contribute to mutation, chromosome instability, cell-cycle arrest, senescence, or cell death. Activation of a DNA-damage-response pathway demonstrates recognition of genomic stress but does not establish successful lesion removal or accurate replication recovery. The final outcome therefore depends on lesion identity and persistence, cell-cycle context, checkpoint competence, and functional repair capacity [176,177,179,180].

#### 6.4.2. Metal-Specific Genotoxicity and Repair Impairment

Pb-associated genotoxicity is predominantly indirect and reflects oxidative imbalance, disruption of thiol-dependent defenses, altered Ca^2+^ signaling, mitochondrial dysfunction, and interference with selected DNA-repair processes rather than extensive direct interaction with DNA. Occupational studies have reported increased strand-break-type damage in exposed lymphocytes, although such findings cannot be attributed specifically to atmospheric PM without source-resolved exposure information. Experimental evidence further indicates that Pb can impair selected repair steps, including inhibition of apurinic/apyrimidinic endonuclease 1 (APE1)-associated AP-site processing, thereby increasing the persistence of repair intermediates. These effects are target-specific rather than evidence of uniform suppression of all DNA-repair pathways [20,166,174,181,182,183].

Cd-associated DNA injury is likewise predominantly indirect, arising through disruption of glutathione and protein-thiol homeostasis, antioxidant defenses, essential-metal regulation, mitochondrial function, and prolonged intracellular retention. Cd can additionally interfere with selected genome-maintenance proteins through interactions with sulfhydryl groups and Zn-dependent structural domains, potentially affecting protein conformation, DNA binding, lesion recognition, and catalytic activity. Experimental inhibition of selected base-excision-repair- and nucleotide-excision-repair-related proteins supports repair impairment as a contributing mechanism, but the affected targets and magnitude of inhibition depend on Cd concentration, exposure duration, cellular model, and intracellular metal handling; these findings should not be generalized to uniform suppression of all repair pathways [21,118,166,175,183,184,185].

Ni-associated genotoxicity is particularly dependent on compound identity, particle solubility, cellular uptake, intracellular dissolution, and the resulting availability of Ni^2+^. Both oxidative DNA injury and interference with genome-maintenance enzymes have been reported, but responses differ substantially among Ni salts, oxides, sulfides, and other particulate forms. Ni^2+^ can inhibit selected Fe^2+^/2-oxoglutarate-dependent enzymes involved in chromatin regulation and DNA repair, while particulate Ni has also been shown experimentally to reduce removal of DNA lesions generated by co-occurring organic carcinogens. This latter finding is especially relevant to PM because it illustrates how one particle constituent may increase the persistence of damage generated by another; nevertheless, results obtained with specific Ni compounds should not be extrapolated to all forms of PM-associated Ni [22,23,119,186,187,188,189].

#### 6.4.3. Interpretation of DNA-Repair and Gene-Based Biomarkers

DNA-repair pathways respond to different classes of genomic lesions and should not be treated as interchangeable indicators of genome maintenance. Base excision repair (BER) primarily processes small, non-helix-distorting lesions such as oxidized bases and AP sites; nucleotide excision repair (NER) removes bulky, helix-distorting lesions; mismatch repair (MMR) corrects replication-associated mismatches; and homologous recombination (HR) and non-homologous end joining (NHEJ) process DNA double-strand breaks. These pathways operate within the broader DNA-damage-response network, in which ATR signaling is associated predominantly with replication stress and ataxia-telangiectasia mutated (ATM) signaling with double-strand-break responses. Activation of a repair or signaling pathway indicates recognition of genomic stress but does not demonstrate that lesions have been removed accurately or that genomic integrity has been restored [177,179,180,190,191,192].

Gene-expression panels can provide useful mechanistic information when selected according to predefined biological pathways, but they should not be interpreted as direct quantitative measures of DNA damage or repair capacity. Representative gene-level targets may include 8-oxoguanine DNA glycosylase 1 (*OGG1*), *APEX1*, and *XRCC1* for BER; *XPA*, *XPC*, and *ERCC1* for NER; *ATM*, *ATR*, *CHEK1*, *CHEK2*, and *TP53* for DNA-damage signaling; and, where integration with oxidative or metal-responsive pathways is required, *NFE2L2*, *HMOX1*, *GCLC*, and *MT2A*. These markers represent functionally distinct processes and should therefore be interpreted as pathway-oriented indicators rather than universally validated or metal-specific biomarkers [172,173,177,179,180,190].

Transcript or protein abundance alone does not establish functional DNA-repair competence. Increased expression may reflect successful adaptation, compensatory induction in response to increased lesion burden, or an insufficient response to persistent damage, whereas unchanged expression does not exclude catalytic inhibition, metal substitution, altered protein localization, defective recruitment to damaged chromatin, or disruption of repair-complex assembly. Gene-based biomarkers are therefore most informative when integrated with functional endpoints such as enzyme activity, pathway-specific repair assays, molecular lesion burden, replication-stress markers, mutation frequency, and chromosome-level outcomes [174,175,177,179,180,183,185,188,189].

### 6.5. Epigenetic and Non-Coding RNA Remodeling

Epigenetic and non-coding-RNA responses provide potential regulatory links between PM-associated metal exposure and persistent changes in cellular phenotype. Relevant mechanisms include DNA methylation, histone modification and chromatin remodeling, and regulation by non-coding RNAs; however, these processes represent distinct regulatory layers and should not be inferred from altered gene expression alone. Likewise, detection of an exposure-associated methylation mark, histone modification, or non-coding-RNA change does not by itself establish causality, functional relevance, or stable epigenetic memory. Interpretation requires consideration of genomic location, cell type, exposure timing, metal persistence, corresponding transcript or protein changes, functional phenotype, and, where persistence is claimed, evidence that the regulatory alteration remains after the initiating exposure or intracellular metal availability has declined [193,194,195,196,197,198,199,200].

#### 6.5.1. DNA Methylation and Chromatin Remodeling

DNA methylation and histone modifications are major, interconnected regulators of chromatin state and transcriptional activity. In mammalian cells, DNA methylation occurs predominantly at cytosine–phosphate–guanine (CpG) dinucleotides and is established or maintained by DNA methyltransferases (DNMTs), whereas ten-eleven translocation (TET)-family dioxygenases contribute to active remodeling of cytosine-modification states. The biological significance of an exposure-associated methylation change depends strongly on genomic location: global or repetitive-element hypomethylation, promoter-associated hypermethylation, and site-specific CpG changes represent distinct regulatory phenomena and should not be interpreted as equivalent indicators of transcriptional activation or repression [193,194,195,201,202].

Histone post-translational modifications and higher-order chromatin organization provide an additional regulatory layer by modifying nucleosome accessibility and recruitment of transcriptional, replication, and DNA-repair proteins. Acetylation and methylation of individual histone residues can be associated with transcriptionally permissive or repressive chromatin states, but their functional meaning depends on the modified residue, genomic location, surrounding chromatin context, and interacting proteins. Accordingly, neither detection of a single histone mark nor a change in chromatin compaction is sufficient to establish transcriptional regulation or impaired DNA repair at a specific locus; epigenetic measurements are most informative when integrated with locus-specific transcriptional, protein, and functional data [180,190,196,197].

#### 6.5.2. Metal-Specific Epigenetic Effects

Pb-associated epigenetic effects are heterogeneous and depend on exposure timing, dose, tissue, developmental stage, and the type of epigenetic endpoint measured. Experimental studies have linked Pb exposure to altered DNA-methyltransferase activity or expression and changes in global DNA methylation, whereas population studies have identified locus-specific methylation associations in blood-derived samples. These findings support the potential for Pb-associated methylation remodeling but do not define a universal Pb-specific epigenetic signature. Moreover, blood-based methylation patterns should not be assumed to reproduce regulatory states in pulmonary, neural, renal, skeletal, or other target tissues, and mechanistic interpretation is strongest when methylation changes are linked to corresponding transcript, protein, or functional phenotypes [200,203,204].

Cd-associated epigenetic remodeling is strongly time- and context-dependent. Experimental models indicate that relatively short Cd exposure may reduce DNA-methyltransferase activity and promote modest genomic hypomethylation, whereas prolonged exposure accompanied by cellular transformation may be associated with increased DNA-methyltransferase activity, increased methylation, and locus-specific gene silencing. These apparently divergent responses emphasize that methylation measured after chronic exposure may reflect not only direct Cd effects but also adaptation, altered proliferation, clonal selection, and survival of cells exposed to persistent intracellular metal. Accordingly, transformation-associated methylation changes should not be generalized to all environmental or PM-associated Cd exposures, and altered DNMT expression alone should not be interpreted as evidence of functionally relevant DNA-methylation remodeling [21,118,199,205,206].

Ni provides particularly strong experimental evidence for chemical-form- and context-dependent chromatin dysregulation. Carcinogenic Ni compounds have been associated with locus-specific transcriptional silencing accompanied by DNA methylation and chromatin condensation, as well as with alterations in histone acetylation and methylation. Ni^2+^ can also inhibit selected Fe^2+/^2-oxoglutarate-dependent chromatin-regulatory enzymes, providing a mechanistic link between intracellular Ni availability and altered chromatin state. For poorly soluble Ni-containing particles, progressive intracellular dissolution may prolong Ni^2+^ availability, but particle persistence, continued metal release, maintenance of an epigenetic mark, mitotic transmission of that mark, and stable epigenetic reprogramming are distinct phenomena and should not be treated as equivalent [23,119,188,207,208].

#### 6.5.3. Non-Coding RNAs, Persistence, and Functional Interpretation

Non-coding RNAs, particularly microRNAs (miRNAs), can modify post-transcriptional gene regulation and thereby influence multiple interconnected stress-response pathways. Altered circulating miRNA profiles have been reported in occupational or environmental PM-exposure studies, including metal-rich particulate exposures; however, such associations do not establish metal specificity or causal involvement in toxicity. Because circulating miRNA abundance depends on blood-cell composition, cellular activation, extracellular release, RNA stability, and sampling time, changes measured in whole blood, plasma, or leukocytes should not be assumed to reproduce regulatory responses in pulmonary, renal, neural, or other target tissues [198,209,210].

Long non-coding RNAs (lncRNAs) represent a heterogeneous regulatory class whose biological significance cannot be inferred from differential expression alone. PM-related studies have identified exposure-associated lncRNA changes, including responses to experimental carbon–metal mixtures and to PM_2.5_, but expression profiling and pathway enrichment primarily generate mechanistic hypotheses. Functional relevance is more strongly supported when altered lncRNA abundance is linked experimentally to a defined molecular target, transcript or protein change, and cellular phenotype; findings obtained with reductionist mixtures should not be attributed automatically to a single metal or generalized to heterogeneous ambient PM [211,212].

Persistence of a regulatory alteration should be distinguished from continued biological response during ongoing exposure or prolonged intracellular metal retention. This distinction is especially important for Cd, which may remain in tissues for years, and for poorly soluble Ni-containing particles that can continue to release Ni^2+^ after deposition. Stable epigenetic memory requires evidence that a regulatory state persists after external exposure has ceased or intracellular metal availability has substantially declined and, where relevant, that it is maintained through subsequent cell divisions while remaining functionally linked to gene expression or phenotype. Particle persistence, tissue metal retention, stable DNA methylation or histone states, persistent non-coding-RNA expression, mitotic transmission, and transgenerational inheritance are therefore separate endpoints and should not be treated as equivalent [21,23,118,119,199,205,206,207,208].

### 6.6. Cytogenetic Damage and Chromosome-Level Biomarkers

Molecular DNA damage, replication stress, inaccurate repair, chromatin dysfunction, and chromosome-segregation errors may ultimately generate abnormalities detectable at the chromosome or nuclear level. Relevant endpoints include structural chromosome aberrations, aneuploidy, sister chromatid exchanges (SCEs), micronuclei (MN), nucleoplasmic bridges (NPBs), and nuclear buds (NBUDs), which represent complementary rather than interchangeable manifestations of genome destabilization. A single increase in one of these endpoints demonstrates chromosome- or nuclear-level damage but does not necessarily establish persistent chromosome instability, which requires evidence of continuing structural or numerical change across successive cell divisions [213,214,215,216,217,218,219].

#### 6.6.1. Chromosome-Level Damage and Micronucleus Formation

Structural chromosome abnormalities may arise when DNA strand breaks or replication-associated lesions persist into mitosis and are processed inaccurately, whereas numerical abnormalities primarily reflect chromosome-segregation failure. These processes can generate chromosome or chromatid breaks, acentric fragments, exchange-type rearrangements, lagging chromosomes, and aneuploid daughter cells. Sister chromatid exchanges represent a related but distinct endpoint reflecting recombinational processing of replication-associated lesions and should not be interpreted as equivalent to chromosome breakage or chromosome loss. For PM-associated Pb, Cd, and Ni, oxidative injury, replication stress, impaired DNA repair, and disruption of chromatin or mitotic processes provide biologically plausible routes to chromosome-level damage, although the relative contribution of each pathway depends on metal form, particle characteristics, intracellular dose, and cell type [180,190,192,214,215,216,218,219].

Micronuclei (MN) are small extranuclear bodies containing chromosomal material that fails to be incorporated into the main daughter nuclei after cell division. They may arise from acentric chromosome or chromatid fragments generated by clastogenic damage or from whole chromosomes or chromatids lost through aneugenic segregation failure. An increase in total MN frequency therefore indicates chromosome-level damage but does not by itself distinguish between breakage and mis-segregation. Mechanistic resolution can be improved by centromeric fluorescence in situ hybridization (FISH) or kinetochore staining, because centromere-negative MN are generally consistent with acentric fragments, whereas centromere-positive MN more often indicate retention of centromeric chromosomal material; this classification nevertheless remains probabilistic rather than absolute [213,214,220].

Human and experimental evidence supports the usefulness of MN as integrative biomarkers of metal-associated genotoxicity, although source attribution remains a major limitation. In environmentally exposed children, blood Pb concentration was associated with increased MN frequency, with both centromere-positive and centromere-negative MN suggesting contributions from segregation-related and breakage-related mechanisms. In cultured human cells, defined Cd and Ni salts increased MN frequency and produced mixed kinetochore-positive and kinetochore-negative responses, supporting both aneugenic and clastogenic potential under the tested conditions. Studies of welders exposed to complex metal-containing aerosols have likewise reported increased micronuclear and DNA-damage endpoints, but such responses cannot be assigned specifically to Pb, Cd, or Ni because welding fumes contain multiple metals and other co-occurring toxicants. Evidence obtained with soluble salts or complex occupational aerosols should therefore be interpreted as mechanistic or biomonitoring support rather than as quantitative proof of effects from a defined PM-bound metal species [221,222,223].

#### 6.6.2. Cytogenetic Biomarkers and PM-Exposure Interpretation

Nucleoplasmic bridges (NPBs) and nuclear buds (NBUDs) provide complementary information to MN in cytokinesis-blocked cells. NPBs may reflect dicentric chromosomes, DNA misrepair, telomere dysfunction, or unresolved chromatin interconnections, whereas NBUDs may indicate abnormal processing or sequestration of excess or amplified chromosomal material. Neither endpoint is metal-specific, and neither should be interpreted mechanistically in isolation. Their value is greatest when assessed together with MN, classical chromosome-aberration analysis, proliferation or cytotoxicity indices, and molecular DNA-damage measurements [213,217].

The cytokinesis-block micronucleus cytome (CBMN-Cyt) assay is particularly useful in biomonitoring because it evaluates MN together with NPBs, NBUDs, cytostasis, cytotoxicity, and nuclear-division parameters in cells that have completed nuclear division. These endpoints represent different temporal and mechanistic levels of genome injury: molecular DNA lesions may be repaired before mitosis and therefore remain undetected as MN, whereas unrepaired lesions, inaccurate repair, or chromosome-segregation defects may become visible only after cell division. Cytogenetic results should therefore be interpreted together with proliferation and viability indices rather than as isolated measures of genotoxicity [213,214]. For standardized in vitro micronucleus testing, Organisation for Economic Co-operation and Development (OECD) Test Guideline 487 provides procedures and cytotoxicity criteria, whereas the broader CBMN-Cyt framework remains multiparametric [213,224].

For PM-associated metal research, cytogenetic biomarkers are most informative when integrated with exposure characterization and internal-dose information. Relevant variables include PM size fraction and elemental composition, metal speciation or bioaccessibility, blood or urinary metal concentrations, co-associated organic genotoxins, oxidative potential, DNA-damage and repair endpoints, inflammatory markers, and the interval between exposure and sampling. Because age, sex, smoking, nutritional status, occupational history, co-exposures, biological matrix, culture conditions, and scoring methodology may also influence cytogenetic outcomes, increased MN, NPB, NBUD, SCE, or chromosome-aberration frequencies should be interpreted as evidence of genome destabilization rather than as specific biomarkers of Pb, Cd, or Ni. Centromeric FISH or kinetochore staining should be incorporated when distinction between predominantly clastogenic and segregation-related mechanisms is required [213,214,220,221,222,223].

Selected molecular, gene-based, epigenetic, and cytogenetic endpoints relevant to PM-associated Pb, Cd, and Ni are summarized in Table 8. These endpoints represent different levels of biological response and should be interpreted as complementary pathway-oriented indicators rather than as interchangeable or metal-specific biomarkers.

### 6.7. Carcinogenicity and Long-Term Disease Susceptibility

The long-term carcinogenic relevance of PM-associated Pb, Cd, and Ni reflects the interaction of exposure characteristics with metal-specific toxicokinetics and multiple biological processes, including persistent oxidative and inflammatory stress, DNA damage and repair impairment, regulatory remodeling, and chromosome-level genome destabilization. These mechanisms may increase carcinogenic susceptibility when damage persists or is propagated in surviving proliferative cells, but no single molecular or cytogenetic endpoint is sufficient to establish malignant transformation. Carcinogenic hazard, mechanistic plausibility, biomarker response, and exposure-specific cancer risk should therefore remain conceptually distinct, particularly for heterogeneous atmospheric PM in which Pb, Cd, and Ni coexist with other inorganic and organic toxicants [17,18,24,25,229,230].

#### 6.7.1. Carcinogenic Hazard in the Context of Atmospheric PM Exposure

Outdoor air pollution and particulate matter in outdoor air pollution are classified by IARC as carcinogenic to humans (Group 1), with sufficient evidence that outdoor air pollution and its particulate component cause lung cancer; limited evidence has also been reported for urinary-bladder cancer in relation to outdoor air pollution. These classifications apply to the complex atmospheric exposure and do not imply that every PM constituent is independently carcinogenic or that Pb, Cd, or Ni individually accounts for the carcinogenic effect of the complete mixture [229,231].

Epidemiological evidence further supports an association between long-term ambient PM exposure and lung cancer risk. In a meta-analysis of 18 studies, Hamra et al. [232] reported a meta-relative risk of 1.09 (95% confidence interval, 1.04–1.14) per 10 µg/m^3^ increase in PM_2.5_ and 1.08 (95% confidence interval, 1.00–1.17) per 10 µg/m^3^ increase in PM_10_. These estimates describe population-level associations with total ambient PM and do not quantify the independent contribution of Pb, Cd, Ni, or another individual particulate constituent [232].

Carcinogenic hazard should be distinguished from exposure-specific risk. IARC classification identifies whether an exposure can cause cancer under some circumstances but does not quantify carcinogenic potency or the probability of cancer at a particular environmental concentration. For metal-containing PM, risk interpretation additionally requires information on particle size and deposition, metal chemical form and solubility, pulmonary bioaccessibility, absorbed and internal dose, exposure duration, co-associated toxicants, and host susceptibility. Consequently, equal total concentrations of Pb, Cd, or Ni in PM cannot be assumed to produce equivalent biologically effective doses or carcinogenic consequences [11,12,17,18,229].

#### 6.7.2. Metal-Specific Carcinogenic Relevance

For Pb, carcinogenic interpretation depends strongly on chemical form. IARC classified inorganic Pb compounds as probably carcinogenic to humans (Group 2A), whereas organic Pb compounds were classified as not classifiable as to their carcinogenicity to humans (Group 3) in the corresponding evaluation. The available mechanistic evidence supports predominantly indirect genome destabilization through oxidative and mitochondrial stress, altered thiol and Ca^2+^ homeostasis, and interference with selected DNA-repair processes rather than extensive direct covalent interaction with DNA. Pb-associated DNA damage, repair inhibition, cytogenetic abnormalities, and methylation changes therefore provide mechanistic plausibility, but none represents a Pb-specific carcinogenic biomarker, and evidence from mixed environmental exposure cannot be attributed automatically to PM-bound Pb alone [17,20,174,182,200,221].

Cadmium and cadmium compounds are classified by IARC as carcinogenic to humans (Group 1), with the human evidence deriving principally from occupational inhalation studies and supporting lung cancer. For PM-associated Cd, however, hazard classification alone does not define exposure-specific risk, because respiratory deposition, particle retention, dissolution, intracellular Cd^2+^ availability, and duration of tissue exposure influence the biologically effective dose. Cd carcinogenicity is mechanistically consistent with prolonged intracellular retention, oxidative and mitochondrial stress, interaction with thiol- and Zn-dependent proteins, interference with selected DNA-repair processes, and time-dependent regulatory remodeling. These mechanisms may interact during chronic exposure, but findings from soluble Cd compounds or transformation models should not be extrapolated quantitatively to all Cd-bearing atmospheric particles [18,21,118,175,205,206].

Carcinogenic interpretation of Ni requires particularly strict specification of chemical form. Nickel compounds are classified by IARC as carcinogenic to humans (Group 1), whereas metallic nickel remains classified as possibly carcinogenic to humans (Group 2B) [233]; the human evidence for nickel compounds includes cancers of the lung and of the nasal cavity and paranasal sinuses. For inhaled Ni-containing PM, carcinogenic relevance depends on the capacity of a defined chemical or particulate form to deliver and maintain intracellular Ni^2+^, because soluble compounds, poorly soluble but progressively dissolving particles, and metallic Ni differ in pulmonary retention and intracellular dose kinetics. Mechanistically, Ni may contribute through oxidative and inflammatory injury, inhibition of selected Fe^2+^/2-oxoglutarate-dependent enzymes, DNA-repair interference, and chromatin dysregulation, while experimental evidence also indicates that some Ni forms can increase the persistence of DNA lesions generated by co-occurring organic genotoxins. These findings are highly relevant to heterogeneous PM but should not be generalized across all Ni compounds or particle types [18,23,119,188,189].

#### 6.7.3. From Mechanistic Evidence to Long-Term Risk Interpretation

Molecular, epigenetic, and cytogenetic effects occupy intermediate biological levels between exposure and clinically manifest disease. Increased DNA-lesion burden, impaired repair, persistent regulatory alterations, MN, or chromosome abnormalities demonstrate biological response or genome destabilization but are not cancer-specific and do not establish that malignant transformation will occur. The biological consequence of an abnormality depends on whether the affected cell repairs the damage, undergoes arrest or death, or survives and propagates the alteration through subsequent divisions. Cytogenetic endpoints should therefore be interpreted as biomarkers of chromosome-level damage and susceptibility rather than as deterministic predictors of cancer [180,213,214,215,230,234].

Long-term susceptibility to PM-associated metal toxicity also extends beyond carcinogenesis and depends on the interaction between repeated external exposure and metal- or particle-specific persistence within the organism. Exposure persistence, toxicokinetic persistence, molecular persistence, and survival of cells carrying stable abnormalities represent related but non-equivalent phenomena. This distinction is particularly important for Pb, which may be remobilized from long-term skeletal stores; Cd, which can remain in tissues for years; and poorly soluble Ni-containing particles, which may provide a continuing local source of Ni^2+^ after pulmonary deposition. Continued biological effects during metal or particle retention should not automatically be interpreted as autonomous molecular or epigenetic memory [20,21,23,118,119].

The most informative interpretation of long-term effects therefore integrates the complete exposure-to-effect continuum: particle composition and size, metal chemical form, respiratory deposition, dissolution and pulmonary bioaccessibility, absorbed and internal dose, target-organ distribution, molecular and inflammatory responses, DNA-damage and repair competence, regulatory remodeling, cytogenetic abnormalities, and their persistence over time. These levels are causally related but do not constitute an obligatory linear sequence; an initial lesion may be repaired, an abnormal cell may be eliminated, and an adaptive response may restore homeostasis. Conversely, repeated or persistent exposure may increase the probability that multiple disturbances coexist and are retained in susceptible tissues or proliferating cells. Accordingly, the long-term risks of PM-associated Pb, Cd, and Ni cannot be inferred from total airborne metal concentration or from any single molecular biomarker but require integration of exposure, toxicokinetic, mechanistic, and outcome-level evidence [11,12,20,21,23,57,119,230].

## 7. Conclusions

PM-associated Pb, Cd, and Ni constitute an interconnected environmental, food-safety, veterinary, and public-health concern whose significance cannot be inferred from total atmospheric metal concentrations alone. Particle size, metal-bearing phase, chemical form, solubility, dissolution kinetics, bioaccessibility, exposure route, internal dose, and target-tissue distribution jointly determine biological relevance and must be considered when comparing sources, environmental reservoirs, exposure pathways, and toxicological outcomes.

The three metals share several downstream effects, including oxidative imbalance, mitochondrial dysfunction, inflammatory signaling, DNA injury, interference with selected genome-maintenance processes, regulatory remodeling, and chromosome-level damage. They are nevertheless not toxicologically interchangeable. Pb is characterized by predominant erythrocyte association, long-term skeletal storage, endogenous remobilization, and prominent neurological, hematological, cardiovascular, and renal toxicity. Cd combines metallothionein-associated sequestration with prolonged hepatic and renal retention, slow elimination, and cumulative proximal tubular toxicity. Ni effects depend particularly on chemical form and exposure route: soluble compounds provide more rapid Ni^2+^ availability, whereas selected poorly soluble particles may persist at respiratory deposition sites and sustain intracellular exposure.

Molecular DNA damage, activation of damage-response pathways, repair impairment, altered gene expression, epigenetic remodeling, cytogenetic damage, and clinically manifest disease represent distinct biological levels rather than obligatory sequential stages. A molecular lesion may be repaired, a regulatory alteration may be reversible, and a damaged cell may undergo arrest or elimination. Long-term susceptibility increases when repeated or persistent exposure permits lesion accumulation, incomplete repair, regulatory dysregulation, chromosome-level abnormalities, and survival of altered proliferative cells. Detection of an isolated molecular, epigenetic, or cytogenetic endpoint should therefore not be interpreted automatically as evidence of persistent disease progression.

Atmospheric deposition does not terminate the exposure pathway. Deposited metals may accumulate in soil, dust, vegetation, water, and sediment; enter crops, forage, feed, livestock, and food products; or return to the atmosphere through erosion and resuspension. Surface soil consequently functions both as a long-term sink and as a potential secondary source connecting atmospheric contamination with direct ingestion, food-chain transfer, and renewed inhalation exposure. Coordinated monitoring should therefore follow the relevant source-to-exposure pathway rather than apply the same matrices and analytical endpoints to every site.

Effective monitoring should integrate total elemental analysis with particle-size information and, where justified, operational fractionation, chemical speciation, and pulmonary bioaccessibility testing and, where relevant to ingested matrices, gastrointestinal bioaccessibility assessment. These endpoints are complementary but not interchangeable. Bioaccessibility does not equal absorption or systemic bioavailability, operational fractions are not discrete chemical species, and environmental occurrence does not by itself quantify internal dose or biological effect. Analytical methods, sample preparation, quality assurance, detection limits, and reporting units must be sufficiently harmonized to permit valid comparison among matrices, studies, and intervention periods.

Risk reduction should target the environmental reservoir and transfer pathway contributing most strongly to exposure. Phytoextraction removes metal only when contaminated biomass is harvested and removed from the site. Phytostabilization and biochar-based treatments primarily reduce mobility, erosion, resuspension, or biological transfer while leaving most of the contaminant inventory in place, whereas microorganism-assisted approaches may support either extraction or stabilization depending on the plant–microorganism–soil–metal system. Immobilization should not be equated with removal, and a short-term reduction in an operationally extractable fraction does not by itself demonstrate durable exposure or risk reduction. Field validation, assessment of transfer into alternative compartments, and multi-season or multi-year monitoring remain essential.

Predictive models can support transfer estimation, hotspot identification, uncertainty analysis, and intervention planning when the model type is matched to the process and decision context. Transfer factors and regression models summarize measured transfer relationships, geostatistical methods characterize spatial patterns, deterministic and probabilistic models support exposure assessment, and machine-learning approaches can represent nonlinear multivariable relationships when supported by representative data and appropriate validation. A modular framework linking atmospheric transport, post-depositional reservoirs, biological transfer, exposure, and intervention is therefore well suited to the multi-scale source-to-effect continuum considered in this review.

Overall, an effective One Health strategy for PM-associated Pb, Cd, and Ni should integrate source characterization, atmospheric transport and deposition, environmental reservoirs, food-chain transfer, internal exposure, mechanistic and cytogenetic biomarkers, remediation, and predictive modeling within a continuous cycle of monitoring, intervention, field verification, and model updating. Such an approach provides a stronger basis for distinguishing hazard from exposure-specific risk, identifying priority pathways, directing interventions, and reducing impacts across environmental, animal, and human health systems.

## Figures and Tables

**Table 1:** Comparative overview of major atmospheric sources, PM-associated characteristics, and source-attribution considerations for Pb, Cd, and Ni.

Characteristic	Pb	Cd	Ni
Major anthropogenic sources	Mining and ore processing; primary and secondary metal production and smelting; lead–acid battery manufacture and recycling; combustion and waste handling; disturbance and resuspension of historically contaminated surfaces; leaded aviation gasoline where still used [20,30,31,38]	Non-ferrous metal production and refining, particularly Zn-associated metallurgy; smelting; fossil-fuel combustion; waste incineration; other high-temperature industrial processes [21,30,31]	Ni mining and refining; stainless-steel and alloy production; electroplating and metal processing; coal and residual-oil combustion; marine-engine emissions; waste incineration and industrial dust [23,30,31,32,39]
Major natural/ geogenic contributions	Weathering and erosion of Pb-bearing geological materials and crustal/mineral dust [28,29]	Weathering and erosion of Cd-bearing ores and mineral materials; geogenic mineral dust [28,29]	Weathering and erosion of Ni-bearing sulfide and lateritic deposits, mafic and ultramafic rocks, and other Ni-bearing geological materials [28,29,40,41]
PM-associated characteristics	Predominantly particle-associated; present in metallurgical aerosols, combustion particles, industrial/mineral dust, and resuspended contaminated soil and road dust. Legacy surface reservoirs may contribute Pb to contemporary atmospheric PM through resuspension [7,20]	High-temperature processes may promote volatilization followed by condensation, adsorption, or surface reaction during cooling, favoring Cd enrichment in fine and submicrometric particles [33,34]	Occurs in multiple chemical forms and particle-size fractions. Combustion-related Ni may be enriched in fine PM, whereas mechanically generated and geogenic Ni can contribute to coarser fractions [23,32,39]
Source indicators/ attribution tools	Pb-isotope ratios, particularly ^206^Pb/^207^Pb and ^208^Pb/^206^Pb, can assist source discrimination and mixing analysis when potential end-members have sufficiently distinct isotopic compositions; interpretation should be integrated with concentrations, spatial patterns, and independent source information [42]	Fine-particle Cd enrichment may support attribution to non-ferrous metallurgy, smelting, waste incineration, and other high-temperature sources when interpreted with co-occurring elements and local source information; Cd alone is not a unique source tracer [30,31,33,34]	Ni–V co-occurrence and V/Ni ratios may support identification of residual-oil or shipping-related contributions when combined with additional tracers and local fuel-use information; they should be regarded as context-dependent indicators rather than unique fingerprints [32,39]
Regional/source-attribution considerations	Relative contributions depend on industrial activity, regulatory history, fuel use, and the extent of legacy contamination. In the United States, leaded aviation gasoline used primarily by small piston-engine aircraft remains a relevant localized source, but this source profile should not be extrapolated to regions with different fuel use and regulatory histories [38]	Relative importance varies with industrial structure, particularly non-ferrous metallurgy, Zn processing, combustion, and waste-management activities. Industrial dust and resuspended contaminated material may complicate source attribution [21,30,31]	Fuel composition, combustion technology, shipping activity, industrial structure, and geological background influence source profiles. Elevated geogenic Ni associated with mafic/ultramafic parent materials may complicate separation of natural and anthropogenic contributions [39,40,41]

**Table 2:** Representative regional PM-associated concentrations and atmospheric deposition metrics for Pb, Cd, and Ni.

Region/Site Setting	Observation/Unit	Pb	Cd	Ni	Interpretation/Reference
Shanghai, China—urban site near a major expressway; 12 selected days, August–September 2014	Total suspended particles concentration, mean ± standard deviation (ng m^−3^)	29.7 ± 6.2	1.8 ± 0.9	10.1 ± 4.3	Short-duration campaign with frequent rainfall; values are site- and period-specific rather than annual urban means [54].
Beijing, China—urban PM_10_ comparison compiled for the Beijing–Tianjin–Hebei region	PM_10_ concentration (ng m^−3^)	157	4	55	Illustrates a relatively high urban PM-associated metal burden; values compiled by Liu et al. from prior Beijing monitoring and should not be treated as contemporaneous with the other rows [55].
Netherlands—rural-background national monitoring, 2023; PM_10_ filter site NL10627	PM_10_ annual mean (ng m^−3^)	2.6	0.1	0.6	Contemporary low-background European example; heavy-metal data capture at this site was <45%, as noted in the monitoring report [56].
Mainland China—regional atmospheric-modeling domain	Fine-mode share of emissions (%)	55	58	Not reported (NR)	Fine-mode particles showed greater transport potential; values are specific to the modeled inventory, region, and atmospheric conditions [48].
Mainland China—regional atmospheric-modeling domain	Mean bulk-deposition flux (mg m^−2^ y^−1^); dry/wet ratio	1.0365; 1.80	0.1850; 2.91	NR	Dry deposition exceeded wet deposition for Pb and Cd in the modeled domain; ratios are not universal removal efficiencies [48].
North America—mixed urban/industrial, rural, and remote monitoring sites	Median wet-deposition flux (mg m^−2^ y^−1^)	0.470	0.035	0.240	Regional medians mask substantial site-to-site variability related to source proximity, site category, and precipitation [9].
Europe—mixed monitoring sites	Median wet-deposition flux (mg m^−2^ y^−1^)	1.400	0.190	0.620	Considerable variation among urban/industrial and less anthropogenically influenced sites [9].
Middle East—mixed monitoring sites, including dust- and anthropogenically influenced locations	Median wet-deposition flux (mg m^−2^ y^−1^)	5.800	0.530	4.900	Highest regional medians among the four regions summarized by Cheng et al.; both crustal and anthropogenic sources may contribute [9].
Asia—mixed monitoring sites	Median wet-deposition flux (mg m^−2^ y^−1^)	3.400	0.140	0.700	Strong spatial variability reflects differences in source intensity, site category, and precipitation [9].

Note: Atmospheric concentration and deposition flux are distinct metrics and should not be interpreted as directly equivalent. Concentration values are reported in ng m^−3^; deposition fluxes are reported in mg m^−2^ y^−1^. Cross-study comparisons should account for PM fraction, sampling period, site type, analytical design, source proximity, meteorology, and precipitation. Regional wet-deposition medians from Cheng et al. [9] integrate heterogeneous monitoring sites and therefore do not represent uniform regional background values. NR, not reported.

**Table 4:** Remediation strategies for post-depositional soil reservoirs of PM-associated Pb, Cd, and Ni.

Strategy	Principal Mechanism/Objective	Relevance to PM-Derived Metal Reservoirs	Key Performance Endpoints	Key Considerations for Application	References
Phytoextraction	Root uptake followed by translocation to harvestable above-ground biomass and physical removal from the site	Can progressively reduce metal inventory in diffusely contaminated surface soils resulting from long-term atmospheric deposition	Shoot biomass; tissue metal concentration; root-to-shoot translocation; cumulative metal mass removed per unit area; residual soil burden; mobile/phytoavailable fractions	High tissue concentration alone does not indicate efficient removal; low biomass, shallow rooting, phytotoxicity, and depletion of readily available pools may limit long-term efficiency. Harvested biomass must be managed as contaminated material.	[80,81,84]
Phytostabilization	Restriction of metal mobility and dispersion through root/rhizosphere retention and persistent vegetation cover	Particularly relevant to reductions in wind erosion, contaminated-dust generation, runoff, and secondary PM resuspension from polluted surface soils	Ground cover; root development; aggregate stability; erosion and dust generation; runoff/leachate metal concentrations; above-ground plant metal concentration; treatment persistence	Does not remove the contaminant inventory; vegetation loss, drought, flooding, fire, overgrazing, cultivation, or land-use change may remobilize retained metals.	[80,81,84]
Chelator-assisted phytoextraction	Chemical mobilization of soil-bound metals to increase root uptake and transfer into harvestable biomass	May increase removal where strongly retained Pb, Cd, or Ni limits conventional phytoextraction	Dissolved/extractable metal; plant biomass; tissue concentration; total metal mass removed; leachate concentration; vertical soil distribution; chelator persistence	Enhanced solubility may increase leaching, groundwater contamination, off-site transport, phytotoxicity, nutrient disturbance, and mobilization of non-target elements. Uptake increase does not necessarily equal risk reduction.	[84,87]
Plant growth-promoting rhizobacteria (PGPR)	Modification of plant growth, stress tolerance, nutrient acquisition, rhizosphere chemistry, biosorption, complexation, or metal mobility	May support vegetation establishment on contaminated deposited-dust/soil reservoirs and can favor either phytoextraction or phytostabilization	Microbial persistence; plant biomass; root/shoot concentrations; rhizosphere pH; mobile metal fractions; translocation; cumulative removal; leaching	Effects are strain-, host-, soil-, and metal-specific. Improved biomass alone does not establish greater remediation. Laboratory/pot results require field validation.	[84,85]
Arbuscular mycorrhizal fungi (AMF)	Extension of root-associated absorptive networks, modification of plant nutrition and stress tolerance, and retention or redistribution of metals in fungal/root/rhizosphere compartments	May enhance vegetation establishment and reduce transfer to shoots in some contaminated-soil systems or increase total uptake in others	Root colonization; extraradical hyphae; biomass; root/shoot metal concentration; translocation; mobile soil fractions; aggregation; plant stress indicators	Outcome depends strongly on fungal isolate, host plant, metal, soil properties, nutrient status, and colonization. Reduced shoot concentration may reflect true retention or simple biomass dilution.	[84,86]
Biochar-assisted immobilization	Sorption, ion exchange, complexation, precipitation, and modification of pH, cation exchange capacity (CEC), organic carbon, and soil structure	Can reduce mobility, plant uptake, runoff or resuspension-related exposure while retaining metals within the treated surface reservoir	Dissolved/exchangeable fractions; porewater/leachate concentrations; plant uptake; soil pH and CEC; dust generation; resuspended-particle metal concentration; multiseason stability	Does not remove total metal inventory. Performance depends on feedstock, pyrolysis, biochar chemistry, soil properties, metal form, aging, redox conditions, and hydrology; fine biochar particles may themselves move off site.	[82,83,84]
Combined plant–microbe–amendment strategies	Integration of vegetation establishment, biological tolerance, uptake or stabilization, and physicochemical immobilization	Potentially useful when multiple pathways—plant transfer, erosion, runoff, and resuspension—must be controlled simultaneously	Metal mass balance; plant uptake; mobile fractions; runoff/leaching; surface stability; dust generation; food/feed transfer; long-term field performance	Interactions may be non-additive; improvement in one endpoint can coexist with deterioration in another. Field-scale durability and transfer to alternative compartments must be demonstrated.	[81,82,83,84]

Note: Remediation endpoints are not interchangeable. Metal removal requires physical export of contaminant mass from the site, whereas immobilization or stabilization reduces mobility or exposure potential while leaving most of the contaminant inventory in place. Changes in operationally extractable fractions should not be equated automatically with changes in pulmonary bioaccessibility, systemic bioavailability, or health risk.

**Table 5:** Representative Pb, Cd, and Ni concentrations in crop matrices from studies evaluating atmospheric deposition or PM-associated exposure.

Country/Exposure Setting	Crop/Edible Organ	Pb (mg/kg)	Cd (mg/kg)	Ni (mg/kg)	Concentration Basis/ Sample Preparation	Main Interpretation Relevant to PM-Associated Transfer	Reference
Netherlands; field exposure vs. dust-free growth chamber	Spinach (*Spinacia oleracea* L. cv. Mazurka), leaves	1.59	1.150	NR	Dry-matter basis; field-grown plants; isotope-labelled soil (^109^Cd/^210^Pb) and dust-free reference chamber	Isotope dilution attributed 73% of spinach Pb to atmospheric deposition. In contrast, no significant airborne contribution to Cd was demonstrated, showing that atmospheric importance can differ markedly between metals in the same crop.	[101]
Netherlands; field exposure vs. dust-free growth chamber	Wheat (*Triticum aestivum* L. cv. Adonis), grain	0.43	0.106	NR	Dry-matter basis; field-grown grain; isotope-labelled soil and dust-free reference chamber	The calculated atmospheric contribution was 100% for Pb and 21.4% for Cd in wheat grain, demonstrating direct atmospheric relevance even for a reproductive edible organ.	[101]
Italy; Castelfiorentino, Tuscany; standardized plants exposed at urban, industrial, and rural sites	Lettuce (*Lactuca sativa* L.), leaves	1.59 ± 0.504	0.59 ± 0.139	3.05 ± 2.241	Dry weight; unwashed lettuce from 11 exposure stations	Lettuce was used as a biodeposimeter of airborne trace elements. Washing significantly reduced several elemental concentrations, indicating substantial surface deposition; source analysis identified anthropogenic contributions, while Cd showed strong non-crustal enrichment. Importantly, these unwashed values include surface-retained material and are not equivalent to a prepared edible matrix.	[102]
Greece; industrial area of Thessaloniki	Lettuce (*Lactuca sativa* L.), leaves	11.2	0.58	NR	Dry weight; median concentrations; n = 8	The original study found relatively low soil contamination but atmospheric PM strongly enriched in Cd and Pb; multivariate analysis indicated that metals in leaves originated predominantly from the atmosphere, with particularly important atmospheric accumulation of Pb and Cd in leafy vegetables.	[103]
France; atmospheric fallout from a Pb-recycling facility	Lettuce (*Lactuca sativa* L.), leaves	335 ± 50	NR	NR	Dry weight; thoroughly washed leaves after 43 days of exposure	Despite thorough washing, very high Pb remained in leaves. Pb-rich particles were identified beneath the leaf surface and submicrometric particles within stomatal openings, providing direct evidence of foliar interaction and internalization of atmospheric Pb-bearing particles.	[104]
France; controlled foliar exposure to Pb-rich process PM from a secondary Pb smelter	Spinach (*Spinacia oleracea* L.), leaves	485.5 ± 14.8	4	NR	Dry weight; leaves thoroughly washed after controlled process-PM exposure	The soil surface was protected from PM, isolating the foliar pathway. Process PM contained particles spanning coarse to submicrometric fractions and was strongly enriched in Pb; substantial Pb and Cd remained in washed spinach leaves after exposure.	[105]
France; same controlled process-PM experiment	Cabbage (*Brassica oleracea* L.), leaves	214.2 ± 5.0	2	NR	Dry weight; leaves thoroughly washed after controlled process-PM exposure	Under the same process-PM exposure, cabbage accumulated less Pb and Cd than spinach, illustrating strong species dependence of foliar PM-associated metal accumulation.	[105]
China; industrial/mining-influenced paddy field, Zhuzhou, Hunan Province	Rice (*Oryza sativa* L.), grain	NR	0.755–1.258	NR	Plants washed with deionized water and dried before analysis; concentration as reported for grain	Five-year monitoring showed atmospheric deposition to be the dominant Cd input pathway at this site: the atmospheric Cd deposition input reached 29.89 g ha^−1^ and represented 96.45% of total Cd input flux. Atmospheric Cd deposition input and soil Cd were both important determinants of Cd accumulation in rice tissues; 96.45% must not be interpreted as the fraction of grain Cd directly derived from atmosphere.	[106]
China; Hebei Province; field source-apportionment study plus flag-leaf exposure experiment	Wheat (*Triticum aestivum* L.), grain	0.33–0.48	NR	NR	Experimental grain concentration after PbSO_4_ flag-leaf exposure; mg/kg as reported	Pb-isotope analysis of field samples showed airborne Pb contributed >50% of grain Pb; estimated atmospheric contributions averaged 78.22% in white flour and 56.27% in bran. In the flag-leaf experiment, PbSO_4_ accounted for 82.80–100% of Pb in exposed grain.	[107]
India; Varanasi; pot cultures exposed at three urban/peri-urban sites	Spinach (*Spinacia oleracea* L.), leaves; Radish (*Raphanus sativus* L.), leaves/root; Tomato (*Lycopersicon esculentum* Mill.), fruit	0.12–14.47	0.078–4.80	0.049–5.06	Edible portions washed to remove surface dust and oven-dried at 80 °C; values originally reported in µg/g	Atmospheric deposition ranged from 20.35–140.00 g ha^−1^ y^−1^ for Pb, 1.77–20.99 for Cd, and 5.45–67.11 for Ni. Site synchrony and air-accumulation factors indicated atmospheric deposition as the main contributor; concentrations were generally highest in leaves, followed by tomato fruit and radish root.	[108]

Note: Concentrations are presented in mg/kg on the concentration basis reported in each original study; 1 µg/g is numerically equivalent to 1 mg/kg. Because the studies differ in concentration basis and sample preparation, values reported on different bases should not be compared directly. The strength of evidence for atmospheric contribution also varies according to study design: Dalenberg and van Driel [101] used isotope-labelled soils together with a dust-free reference environment; Nali et al. [102] used standardized lettuce as a biodeposimeter of airborne trace elements; Voutsa et al. [103] compared vegetables, soils, and airborne particulate matter; Uzu et al. [104] examined direct exposure to industrial atmospheric fallout; Xiong et al. [105] used controlled foliar exposure to PM while preventing soil contamination; and Liu et al. [106], Zhang et al. [107], and Pandey et al. [108] quantified atmospheric inputs or applied source-attribution approaches in crop systems. For Voutsa et al. [103], the lettuce concentrations and source interpretation derive from the original study. The 0.33–0.48 mg/kg Pb range reported by Zhang et al. [107] refers to grain obtained in the experimental PbSO_4_ flag-leaf exposure and does not represent the concentration range observed in the field survey. The ranges reported for Pandey et al. [108] encompass different edible organs and three exposure sites and should not be interpreted as species-specific concentration ranges. The values reported by Xiong et al. [105] derive from a controlled high-exposure experiment and should not be compared directly with concentrations measured in unmanipulated field-grown food crops. NR, not reported or not analyzed for the selected matrix.

**Table 6:** Representative Pb, Cd, and Ni concentrations and tissue-specific accumulation patterns in freshwater and marine fish and selected bivalves relevant to downstream food-chain transfer.

Aquatic System/ Region	Organism/Matrix	Pb (mg/kg)	Cd (mg/kg)	Ni (mg/kg)	Concentration Basis	Main Interpretation Supported by the Study	References
Freshwater—Lake Burgas and Lake Mandra, Bulgaria	Crucian carp (*Carassius carassius*), common carp (*Cyprinus carpio*), roach (*Rutilus rutilus*), Prussian carp (*Carassius gibelio*), and freshwater bream (*Abramis brama*); edible muscle	0.15–0.27	0.02–0.05	0.06–0.11	Wet weight	Species-dependent differences were observed in edible muscle. Prussian carp showed the highest mean Pb concentration (0.27 mg/kg), whereas roach showed the highest mean Cd (0.05 mg/kg) and Ni (0.11 mg/kg). Individual target hazard quotient values and the combined hazard index were <1 under the consumption assumptions used in the study.	[142]
Freshwater—Lower Mississippi River Basin, USA	Channel catfish (*Ictalurus punctatus*); muscle and liver	Muscle: 0.18; liver: 0.56	Muscle: 0.01; liver: 0.16	Muscle: 0.85; liver: 1.11	Wet-weight tissue; concentrations reported as mg/kg	Cd and Pb were substantially higher in liver than muscle; the study found significant muscle–liver differences for Cd and Pb in channel catfish, whereas the Ni difference was not statistically significant. The data therefore demonstrate metal- and tissue-specific accumulation rather than a uniform tissue pattern.	[143]
Marine—Tenerife, Canary Islands, Spain; North-Eastern Atlantic	Salema (*Sarpa salpa*); muscle	0.03 ± 0.01	0.007 ± 0.01	0.12 ± 0.33	Wet weight	All three selected metals were quantified in edible muscle. The study found substantially greater concentrations of Cd and Pb, and generally greater concentrations of trace elements, in liver than muscle, emphasizing the importance of the tissue analyzed for dietary interpretation.	[144]
Marine—Gela, Sicily, Southern Italy	Red mullet (*Mullus barbatus*); muscle, two size groups	0.31 ± 0.05 to 0.41 ± 0.01	0.37 ± 0.05 to 0.50 ± 0.01	0.27 ± 0.04 to 0.41 ± 0.05	Wet weight	The larger fish group had higher mean Pb, Cd, and Ni concentrations than the smaller group, supporting a size-associated difference in metal accumulation under the conditions of this study.	[145]
Marine—South China Sea	29 wild marine fish species	0.00054–0.02731	0.00051–0.11581	0.00832–0.05748	Wet weight	Wide interspecies ranges were observed for all three selected metals. The authors’ health-risk assessment did not indicate significant adverse health effects associated with consumption under the assumptions applied in that study.	[146]
Marine—Black Sea coast, Bulgaria	Striped venus clam (*Chamelea gallina*); edible soft tissue, four seasons	0.03–0.55	0.22–0.91	0.03–0.51	Wet weight	Strong seasonal variation was observed: the highest mean concentrations of Pb, Cd, and Ni in *C. gallina* occurred in winter (0.55, 0.91, and 0.51 mg/kg, respectively). The study assessed these concentrations directly in edible tissue.	[147]
Marine—Algerian coast	Brown mussel (*Perna perna*) and Mediterranean mussel (*Mytilus galloprovincialis*); gill, digestive gland, gonad, and remaining soft tissues across sites and seasons	0.10–17.15	0.10–2.60	0.36–25.70	Dry weight	The digestive gland, followed by the gills, showed significantly high metal concentrations, whereas the gonad generally contained lower levels. The ranges integrate different tissues, species, sites, and seasons and therefore must not be treated as standardized edible-tissue concentrations.	[139]

Note: Concentrations are presented in mg/kg on the concentration basis reported in each original study; 1 µg/g is numerically equivalent to 1 mg/kg. Wet- and dry-weight concentrations should not be compared directly. The table deliberately distinguishes freshwater fish from marine fish and includes selected marine bivalves to illustrate differences among aquatic systems, taxa, and tissues. For finfish, muscle represents the principal edible matrix considered in dietary exposure assessments, whereas liver, gills, and other metabolically active tissues may exhibit substantially different accumulation patterns. Bivalves may integrate dissolved, particulate, and dietary exposure and therefore should not be compared directly with fish muscle. Ranges derived across multiple species, size groups, tissues, sites, or seasons are identified explicitly and should not be interpreted as universal species-specific concentrations. These studies demonstrate aquatic occurrence and bioaccumulation relevant to downstream food-chain transfer but do not, by themselves, establish atmospheric PM as the predominant source of the measured Pb, Cd, or Ni.

**Table 7:** Representative modeling approaches for PM-associated Pb, Cd, and Ni across the source-to-exposure pathway.

Model/ Approach	Model Type and Typical Domain	Primary Application	Key Inputs	Principal Outputs	Added Value and Key Considerations for Application	References
AERMOD	Steady-state atmospheric dispersion model Typical domain: Local to near-source; hourly meteorological resolution with short- and long-term averaging periods	Near-source dispersion of emissions from defined stationary sources and estimation of receptor-level concentrations	Emission rate, source geometry and release characteristics, meteorological data, terrain, land-surface parameters, receptor locations	Predicted ambient concentrations, spatial concentration fields, source-specific receptor impacts	Provides an established framework for evaluating dispersion from defined emission sources and linking source characteristics with downwind concentrations Application considerations: most appropriate for well-characterized near-source applications. Reliable implementation requires representative emissions and meteorological data, appropriate receptor placement, and adequate representation of terrain and source configuration. Regional transport and atmospheric transformation may require complementary modeling approaches.	[68]
HYSPLIT	Hybrid Lagrangian–Eulerian trajectory, transport, dispersion, and deposition model Typical domain: local to regional, continental, or global; hours to days or longer depending on the application	Forward and backward trajectories, source–receptor analysis, atmospheric transport, dispersion, and wet/dry deposition	Source location and release time, meteorological fields, release height, particle characteristics, settling velocity and deposition parameters	Air-mass trajectories, concentration fields, transport pathways, dispersion patterns, and deposition estimates	Particularly useful for investigating transport pathways and potential source–receptor relationships over a wide range of spatial scales Application considerations: trajectory and dispersion results are most informative when integrated with emission inventories, chemical or elemental measurements, meteorological interpretation, and observational data. Particle-size and deposition parameters should reflect the material being modeled.	[69,70]
CMAQ	Eulerian regional chemical-transport model Typical domain: regional to continental; episodic, seasonal, annual, or multiyear applications	Regional simulation of atmospheric transport, chemical transformation, particulate composition, and wet and dry deposition	Emission inventories, meteorological fields, initial and boundary conditions, atmospheric chemistry, land-surface information, particle-size representation, deposition parameters	Spatially and temporally resolved atmospheric concentrations and deposition; PM composition and, in appropriate configurations, source-sensitivity or source-apportionment information	Integrates emissions, meteorology, atmospheric processes, PM composition, and deposition within a common regional modeling framework and can represent particulate toxic metals including Pb, Cd, and Ni Application considerations: application requires internally consistent emission inventories and meteorology, appropriate spatial and temporal resolution, and evaluation against observational measurements. The representation of metal emissions and their distribution among particle modes should correspond to the intended application.	[71,72]
Deterministic exposure model	Point-estimate mathematical exposure model Typical domain: defined individual or population exposure scenario; short-term or chronic depending on input assumptions	Screening-level or scenario-specific estimation of inhaled, ingested, deposited, absorbed, or other route-specific doses and associated risk metrics	Environmental concentration, inhalation or intake rate, exposure frequency and duration, body weight, particle deposition or bioaccessibility where included	Point estimates of exposure, dose, hazard quotient, margin of exposure, or other predefined metrics	Transparent and readily reproducible; useful for screening calculations, comparison of exposure scenarios, and identification of influential assumptions Application considerations: input values should correspond to the population, exposure route, particle fraction, averaging period, and concentration basis being evaluated. Conservative assumptions should be identified explicitly and interpreted according to the purpose of the assessment.	[164]
Soil–plant transfer factor (TF)	Empirical concentration-ratio model Typical domain: site-, soil-, crop-, organ-, and sampling-period specific	Comparison or estimation of metal transfer from contaminated soil to edible or vegetative plant tissues	Paired soil and plant metal concentrations reported on compatible concentration bases	Transfer factor, commonly expressed as TF=Cplant/Csoil	Simple and transparent index for comparative evaluation of soil-to-plant transfer among metals, crops, sites, or environmental conditions Application considerations: interpretation should specify whether total or operationally extractable soil concentration is used and account for soil pH, organic matter, texture, metal availability, crop species or cultivar, plant organ, growth stage, and irrigation conditions.	[153,154,155]
Multivariable regression	Empirical statistical predictive model Typical domain: local or regional according to the calibration dataset	Prediction of environmental, plant, food, or other receiving-matrix concentrations from multiple explanatory variables	Metal concentrations and relevant environmental or biological covariates such as pH, organic matter, texture, extractability, crop characteristics, irrigation, or land use	Predicted concentrations, transfer relationships, regression coefficients, uncertainty or goodness-of-fit statistics	Allows simultaneous consideration of several factors controlling metal mobility and transfer and can improve prediction compared with a single concentration ratio Application considerations: predictor selection should be biologically and environmentally justified, and model performance should be evaluated within the range of conditions represented by the calibration data. Independent validation is desirable when predictions are transferred to new sites or populations.	[153,154]
Kriging/geostatistical modeling	Spatial statistical modeling Typical domain: local to regional depending on sampling design and spatial autocorrelation	Interpolation of metal concentrations, delineation of spatial gradients and hotspots, and prediction at unsampled locations	Georeferenced measurements, sampling geometry, spatial covariance or semivariogram structure, and optionally relevant spatial covariates	Predicted concentration surfaces, spatial gradients, hotspot maps, and model-based prediction variance	Explicitly uses spatial dependence in measured data and is particularly valuable for visualizing contamination patterns and prioritizing additional sampling Application considerations: sampling density, spatial coverage, anisotropy, trends, scale of spatial variation, and the selected covariance or semivariogram model should be evaluated. Spatial predictions are strengthened when interpreted together with source, land-use, and environmental information.	[156]
Probabilistic/Monte Carlo modeling	Stochastic simulation approach Typical domain: population- or scenario-specific; applicable to acute or chronic assessments	Characterization of distributions of exposure or risk and propagation of variability and uncertainty in model inputs	Probability distributions for concentrations, exposure factors, physiological variables, bioaccessibility, or other relevant parameters	Exposure or risk distributions, percentiles, confidence or probability intervals, and probabilities of exceeding predefined thresholds	Provides a distribution of possible outcomes rather than a single point estimate and allows population variability and model uncertainty to be examined explicitly Application considerations: selection of probability distributions, parameter dependence and correlations, sample size, treatment of upper-tail values, and separation of variability from uncertainty should be documented according to the assessment objective.	[157]
Sensitivity analysis	Local or global parameter-influence analysis Typical domain: applicable across atmospheric, environmental-transfer, exposure, and intervention models	Identification of inputs and assumptions exerting the greatest influence on model predictions	Defined model structure together with parameter values, ranges, or probability distributions	Sensitivity coefficients, rankings, variance-based indices, or other measures of parameter influence	Helps identify dominant determinants of predictions and can guide monitoring, experimental design, and collection of additional data Application considerations: the selected sensitivity method should correspond to the model structure and assessment objective. Parameter ranges, correlations, nonlinear relationships, and interactions among inputs should be considered when interpreting parameter importance.	[158]
Machine-learning models	Data-driven predictive models capable of representing nonlinear relationships and interactions Typical domain: determined primarily by the domain represented in the training and validation datasets	Prediction of metal transfer, spatial contamination, bioaccessibility, immobilization efficiency, remediation performance, or other defined outcomes	Environmental, soil, particle, chemical, biological, exposure, or intervention-related predictor variables	Predicted continuous or categorical outcomes, prediction errors, variable-importance metrics, and model-specific explanatory outputs	Can represent complex nonlinear relationships and interactions that may be difficult to specify a priori in conventional statistical models Application considerations: reliable application requires a clearly defined prediction target, representative and internally consistent datasets, appropriate separation of training and test data, independent or spatially structured validation where appropriate, and explicit definition of the model’s applicability domain.	[159,160,161,162]
Modular integrated modeling framework	Linked process-model architecture Typical domain: multiscale; spatial and temporal resolution may differ among linked modules	Integration of atmospheric transport and deposition, environmental reservoirs, transfer pathways, exposure, and intervention scenarios	Outputs from atmospheric, environmental-transfer, exposure, toxicokinetic, or intervention modules together with harmonized boundary conditions and units	Integrated scenario predictions linking changes at one stage of the source-to-exposure pathway with downstream environmental or exposure outcomes	Allows individual processes to be represented using models suited to their own spatial scale and mechanistic requirements while maintaining an integrated source-to-exposure framework Application considerations: before transferring outputs between modules, compatibility should be verified for spatial and temporal resolution, environmental matrix, particle or chemical fraction, concentration basis, units, population definition, boundary conditions, and uncertainty characterization.	[73,163]

Abbreviations: AERMOD, American Meteorological Society/Environmental Protection Agency Regulatory Model; CMAQ, Community Multiscale Air Quality modeling system; HYSPLIT, Hybrid Single-Particle Lagrangian Integrated Trajectory model; PM, particulate matter; TF, soil-to-plant transfer factor. The approaches summarized here address different stages of the source-to-exposure pathway and are therefore complementary rather than interchangeable. Model selection should be guided by the assessment objective, spatial and temporal scale, available data, and intended domain of application.

**Table 8:** Selected molecular, gene-based, epigenetic, and cytogenetic endpoints relevant to genome instability associated with PM-bound Pb, Cd, and Ni.

Domain/Pathway	Representative Markers or Endpoints	Biological Information Provided	Representative Relevance to Pb, Cd, and Ni	Key Considerations for Interpretation	References
Base excision repair (BER)	*OGG1*, *APEX1/APE1*, *XRCC1*, *PARP1*, DNA polymerase β, DNA ligase III	Recognition and processing of oxidized bases, AP sites, and associated single-strand-break intermediates	Pb can inhibit selected repair steps, including APE1-associated AP-site processing; Cd can interfere with selected BER-related proteins through thiol binding and disruption of metal-dependent protein structure. Oxidative DNA injury generated during Pb-, Cd-, or Ni-associated stress may increase demand on BER.	Transcript or protein abundance does not establish catalytic repair capacity; inhibition of one BER component does not demonstrate global BER failure.	[174,175,177,183]
Nucleotide excision repair (NER)	*XPA*, *XPC*, *ERCC1*, *ERCC2*, *ERCC5*	Recognition and removal of bulky, helix-distorting DNA lesions	Cd has experimentally interfered with selected NER-related proteins, including XPA under defined conditions. Ni compounds can also interfere with XPA-related damage recognition and may increase the persistence of lesions produced by co-associated organic genotoxins.	Individual NER genes are not interchangeable measures of pathway activity; soluble-metal experiments should not be extrapolated quantitatively to PM-bound forms.	[175,183,189,190]
Mismatch repair (MMR)	*MSH2*, *MSH6*, *MLH1*, *PMS2*	Recognition and processing of replication-associated mismatches and selected replication errors	Useful as pathway-oriented markers when metal-associated replication stress or genome-maintenance dysfunction is investigated.	The manuscript does not establish a validated Pb-, Cd-, or Ni-specific MMR signature; transcript abundance alone is insufficient evidence of functional MMR impairment.	[180,191]
Homologous recombination (HR)	*BRCA1*, *PALB2*, *BRCA2*, *RAD51*, *MRE11–RAD50–NBS1* complex	High-fidelity processing of DNA double-strand breaks using homologous sequence information, particularly during S/G_2_	Relevant downstream of persistent oxidative lesions, replication-fork collapse, and DSB formation potentially associated with metal-induced genome stress.	Pathway activation does not demonstrate accurate repair; altered expression of HR genes is not a direct measure of mutation or chromosome-aberration frequency.	[180,192]
Canonical non-homologous end joining (c-NHEJ)	*Ku70–Ku80*, *DNA-PKcs*, *XRCC4*, *XLF*, DNA ligase IV	Rejoining of DNA double-strand breaks without an extended homologous template	Potentially relevant to conversion of persistent metal-associated DNA injury into chromosome rearrangements when DSB processing is inaccurate.	Expression of *NHEJ* components does not determine repair fidelity; inaccurate joining must be demonstrated through functional or chromosome-level endpoints.	[192,216]
Direct reversal of DNA lesions	*MGMT*, *ALKBH2*, *ALKBH3*	Direct enzymatic reversal of selected chemically modified bases	Ni^2+^ experimentally inhibits the Fe^2+^/2-oxoglutarate-dependent repair dioxygenase ALKBH2 through catalytic-metal substitution, linking intracellular Ni availability with inhibition of a defined repair enzyme.	*ALKBH2* inhibition is lesion- and enzyme-specific and should not be generalized to all DNA-repair proteins or all Ni compounds.	[188,225]
DNA-damage response and checkpoint signaling	*ATM*, *ATR*, *CHEK1*, *CHEK2*, *TP53*, *CDKN1A*, *GADD45A*	Recognition of DNA damage or replication stress, checkpoint activation, cell-cycle arrest, repair coordination, senescence, or cell-death signaling	Potentially responsive across Pb-, Cd-, and Ni-associated genome stress, depending on lesion class, intracellular dose, and cellular context.	Activation of DNA-damage response (DDR) signaling demonstrates recognition of stress but not successful lesion removal or restoration of genomic integrity.	[179,180]
Oxidative and metal-adaptive response	*NFE2L2/NRF2*, *HMOX1*, *GCLC*, *GCLM*, *MT2A*	Antioxidant response, glutathione metabolism, electrophile defense, and metal sequestration/homeostasis	Relevant across metal-associated oxidative stress; MT2A and metallothionein responses are especially important for Cd sequestration and persistence.	Induction may indicate successful adaptation, compensatory signaling, or an insufficient response during continuing toxicity; it is not synonymous with protection.	[118,172,173]
DNA-methylation machinery	*DNMT1*, *DNMT3A*, *DNMT3B*, *TET1*; global, repetitive-element, promoter, and site-specific CpG methylation	Maintenance, establishment, and remodeling of DNA-methylation states	Pb: altered DNMT activity/expression and global or locus-specific methylation have been reported. Cd: short and chronic exposures may produce temporally different methylation responses; chronic transformation models show increased DNMT3B and promoter-associated methylation.	Global hypomethylation, promoter hypermethylation, and individual CpG changes are biologically distinct. Altered DNMT abundance does not prove functionally relevant methylation remodeling.	[193,194,195,200,203,204,205,206]
Histone modification and chromatin state	Histone acetylation; H3K4me3; H3K9me2/3; H3K27me3; H2A/H2B ubiquitination; KDM3A/JMJD1A	Chromatin accessibility, transcriptional regulation, recruitment of replication and DNA-repair proteins	Ni provides particularly strong experimental evidence: Ni exposure has been associated with chromatin condensation, decreased histone acetylation, increased H3K9 dimethylation and H2A/H2B ubiquitination; Ni^2+^ can inhibit KDM3A/JMJD1A.	A single histone mark cannot establish activation or repression of a defined gene; effects are locus-, compound-, cell-, and exposure-dependent.	[188,196,197,207,208]
Pb-associated epigenetic remodeling	DNMT activity; global methylation; locus-specific CpG methylation	Exposure-associated DNA-methylation changes	Experimental Pb exposure altered DNMT activity/expression and global methylation; human studies identified blood-based locus-specific associations, including early-life and cumulative-exposure patterns.	No universal Pb-specific methylation signature has been established; blood methylation should not be assumed to represent neural, pulmonary, renal, or skeletal tissues.	[200,203,204]
Cd-associated epigenetic remodeling	DNMT activity; DNMT3B; global and promoter-associated methylation	Time-dependent methylation remodeling during prolonged Cd exposure	Shorter Cd exposure produced reduced DNMT activity and modest hypomethylation in one transformation model, whereas prolonged exposure/transformation was associated with increased DNMT activity, increased methylation, DNMT3B induction, and selected gene silencing.	Transformation-associated changes may reflect direct Cd effects, adaptation, altered proliferation, clonal selection, and persistent intracellular exposure and should not be generalized to environmental PM exposure.	[199,205,206]
Ni-associated epigenetic remodeling	DNA methylation, chromatin condensation, histone acetylation/methylation/ubiquitination, *KDM3A/JMJD1A*	Locus-specific transcriptional silencing and chromatin dysregulation	Carcinogenic Ni compounds produced reporter-gene silencing associated with DNA methylation and chromatin condensation; NiCl_2_ altered multiple histone modifications; Ni^2+^ inhibited *KDM3A/JMJD1A*.	Particle persistence, Ni^2+^ availability, maintenance of a histone or methylation state, mitotic inheritance, and irreversible epigenetic reprogramming are separate phenomena.	[188,199,207,208]
MicroRNA responses	Exposure-associated miRNA profiles in leukocytes or circulating compartments	Post-transcriptional regulation and exposure-responsive changes in gene-expression networks	Metal-rich occupational PM exposure was associated with leukocyte miRNA changes and selected Pb/Cd exposure metrics; personal PM_2.5_ exposure was associated with altered circulating miRNA profiles.	MiRNA responses in mixed PM exposures are not necessarily metal-specific; whole blood, leukocytes, plasma, serum, and extracellular vesicles are not interchangeable biological matrices.	[198,209,210]
Long non-coding RNAs	lncRNA expression profiles; NONHSAT074301.2 as a representative experimentally tested candidate	Potential regulation of transcription, cell-cycle responses, and other exposure-associated phenotypes	A carbon nanoparticle–Cr(VI)–Pb(II) mixture induced G2/M arrest and altered NONHSAT074301.2 with functional evidence for involvement; PM_2.5_ exposure has also generated differential lncRNA profiles in experimental cells.	Expression profiling or pathway enrichment alone does not establish causal regulatory function; mixed-exposure studies cannot be attributed specifically to Pb.	[211,212]
Persistence/epigenetic memory	Persistence of DNA methylation, histone state, ncRNA expression, or associated phenotype after exposure withdrawal	Distinguishes transient exposure responses from potentially stable regulatory states	Particularly relevant to Cd because of prolonged tissue retention and to poorly soluble Ni particles because continuing dissolution can sustain intracellular Ni^2+^ availability.	Toxicokinetic persistence is not equivalent to epigenetic memory. Stable memory requires persistence after the initiating exposure or intracellular metal availability has declined; mitotic and transgenerational inheritance represent still higher evidentiary levels.	[199,205,206,207,208]
Chromatid and chromosome breaks	Chromatid breaks; chromosome breaks; acentric fragments	Structural chromosome injury arising from persistent or inaccurately processed DNA lesions	Biologically plausible downstream endpoints of Pb-, Cd-, and Ni-associated oxidative injury, replication stress, and repair interference.	Chromatid- versus chromosome-type patterns are mechanistically informative but should not be treated as exact molecular timestamps of lesion induction.	[190,192,215,216]
Structural chromosome rearrangements	Dicentric chromosomes, ring chromosomes, translocations and other exchange-type rearrangements	Erroneous joining of chromosome segments or dysfunctional chromosome ends	May arise when persistent chromosome breaks are processed inaccurately; dicentrics may produce anaphase or nucleoplasmic bridges and contribute to breakage–fusion–bridge-type instability.	Not every chromatin bridge is produced by a dicentric chromosome; unresolved replication or recombination intermediates can produce similar structures.	[213,216,217]
Chromosome-segregation abnormalities	Nondisjunction, anaphase lag, lagging chromosomes, chromosome gain/loss, aneuploidy	Numerical chromosome damage and segregation failure	Defined Cd and Ni salts have produced segregation-related responses experimentally; environmental Pb-associated MN patterns also suggest a possible aneugenic component.	Segregation failure may involve spindle, centrosome, kinetochore attachment, cohesion, or checkpoint dysfunction and cannot be assigned to one molecular mechanism from aneuploidy alone.	[214,218,221,222]
Micronuclei (MN)	Total MN; centromere-negative MN; centromere/kinetochore-positive MN	Integrated endpoint of chromosome fragmentation and/or segregation failure	Environmental Pb exposure in children was associated with both centromere-negative and -positive MN. Defined Cd and Ni salts increased MN in human fibroblasts, with a substantial kinetochore-positive component. Metal-containing welding-fume exposures have also been associated with increased MN-related damage.	Total MN frequency alone cannot distinguish clastogenic from aneugenic mechanisms. Centromeric FISH or kinetochore staining improves but does not provide absolute mechanistic classification.	[213,220,221,222,223]
Nucleoplasmic bridges (NPBs)	NPBs in binucleated cells	Potential consequence of dicentrics, DNA misrepair, telomere dysfunction, or unresolved chromatin interconnections	Increased NPB frequencies have been reported in environmentally exposed populations, welders, and Pb-treated human lymphocytes.	NPBs are not metal-specific and do not invariably demonstrate dicentric chromosomes.	[213,217,226,227,228]
Nuclear buds (NBUDs)	NBUD frequency	Abnormal sequestration or elimination of excess, amplified, or incompletely resolved DNA-containing material	Increased NBUDs have been reported in environmental/occupational metal-exposure studies and following defined Pb exposure in vitro.	NBUDs have relatively low mechanistic specificity and should not be interpreted as direct evidence of gene amplification without molecular confirmation.	[213,226,227,228]
Sister chromatid exchanges (SCEs)	SCE frequency	Reciprocal exchange between sister chromatids, reflecting recombinational processing of replication-associated lesions	Provides a sensitive complementary endpoint where metal-associated replication stress and recombination are investigated.	SCEs generally do not produce immediate net gain or loss of genetic material and should not be equated with chromosome breaks, dicentrics, MN, or aneuploidy.	[192,215,219]
Integrated CBMN-Cyt phenotype	MN, NPB, NBUD, mono-/bi-/multinucleated-cell distribution, nuclear-division indices, apoptosis/necrosis and cytotoxicity endpoints	Multiparametric assessment of chromosome-level injury in cells completing nuclear division	Useful in environmental and occupational biomonitoring of complex metal-containing exposures and for integrating chromosome damage with proliferation and cytotoxicity.	Cytostasis may prevent damaged cells from reaching mitosis, whereas severe cytotoxicity may remove highly damaged cells; tissue, sampling time, scoring criteria, and exposure mixture strongly affect interpretation.	[213,223,224,226,227]

Abbreviations: BER, base excision repair; CBMN-Cyt, cytokinesis-block micronucleus cytome assay; c-NHEJ, canonical non-homologous end joining; DDR, DNA-damage response; DSB, double-strand break; HR, homologous recombination; lncRNA, long non-coding RNA; MMR, mismatch repair; MN, micronucleus/micronuclei; NBUD, nuclear bud; NER, nucleotide excision repair; NPB, nucleoplasmic bridge; SCE, sister chromatid exchange. Gene and protein markers listed in the table are pathway-oriented candidates or experimentally investigated targets and should not be interpreted as universally validated biomarkers specific to Pb, Cd, Ni, or PM exposure.

## Data Availability

No new data were created or analyzed in this study. Data sharing is not applicable to this article.

## Abbreviations

8-oxoG	8-Oxo-7,8-dihydroguanine; oxidatively modified guanine lesion that may mispair with adenine during DNA replication
AERMOD	American Meteorological Society/Environmental Protection Agency Regulatory Model
AMF	Arbuscular mycorrhizal fungi; symbiotic root fungi that extend soil exploration and may modify metal uptake, translocation, and retention
AP	Apurinic/apyrimidinic; describes a DNA site from which the purine or pyrimidine base is absent
APE1	Apurinic/apyrimidinic endonuclease 1; a base-excision-repair enzyme that cleaves DNA at AP sites
ATM	Ataxia-telangiectasia mutated; a protein kinase involved predominantly in signaling DNA double-strand breaks
ATR	Ataxia telangiectasia and Rad3-related; a protein kinase involved predominantly in replication-stress signaling
ATSDR	Agency for Toxic Substances and Disease Registry
BCR	Community Bureau of Reference; operational sequential-extraction procedure for assessing metal fractionation and potential mobility
BER	Base excision repair; DNA-repair pathway that processes small, non-helix-distorting base lesions and AP sites
BW	Body weight; denominator used to express body-weight-normalized exposure
c-NHEJ	Canonical non-homologous end joining; principal Ku- and DNA-PK-dependent NHEJ pathway
CBMN-Cyt	Cytokinesis-block micronucleus cytome assay; assay evaluating micronuclei and complementary nuclear abnormalities in cells that have completed nuclear division
CEC	Cation exchange capacity; soil capacity to retain and exchange positively charged ions
CMAQ	Community Multiscale Air Quality modeling system
CpG	Cytosine–phosphate–guanine dinucleotide; genomic sequence context in which cytosine methylation commonly occurs in mammalian DNA
CUL3	Cullin 3; scaffold protein of the E3 ubiquitin-ligase complex involved in NRF2 regulation
DDR	DNA damage response; signaling network coordinating lesion recognition, checkpoint activation, DNA repair, cell-cycle control, senescence, and apoptosis
DE	Dietary exposure; contaminant intake normalized, where appropriate, to body weight and time
DNMT	DNA methyltransferase
DSB	Double-strand break; discontinuity affecting both strands of the DNA molecule
EEA	European Environment Agency
EFSA	European Food Safety Authority
EU	European Union
FAAS	Flame atomic absorption spectrometry; element-specific analytical technique using flame atomization
FAO	Food and Agriculture Organization of the United Nations
FISH	Fluorescence in situ hybridization; cytogenetic method using fluorescent probes to detect defined DNA sequences
GFAAS	Graphite furnace atomic absorption spectrometry; trace-element technique using electrothermal atomization
GSH	Reduced glutathione; major low-molecular-weight intracellular thiol and redox buffer
GSSG	Oxidized glutathione disulfide; oxidized form generated from GSH
HR	Homologous recombination; relatively high-fidelity DNA double-strand-break repair pathway using homologous sequence information
HYSPLIT	Hybrid Single-Particle Lagrangian Integrated Trajectory model
IARC	International Agency for Research on Cancer
ICP-MS	Inductively coupled plasma mass spectrometry; highly sensitive multi-element analytical technique based on mass-to-charge detection
ICP-OES	Inductively coupled plasma optical emission spectrometry; multi-element analytical technique based on element-specific optical emission
IR	Ingestion rate; daily amount of a specified food consumed in the dietary-exposure equation
JECFA	Joint FAO/WHO Expert Committee on Food Additives
KEAP1	Kelch-like ECH-associated protein 1; cytoplasmic regulator that targets NRF2 for ubiquitination and degradation under basal conditions
lncRNA	Long non-coding RNA; non-protein-coding transcript with context-dependent regulatory functions
miRNA	MicroRNA; short non-coding RNA that regulates messenger RNA stability or translation
MMAD	Mass median aerodynamic diameter; aerodynamic diameter at which half of the particle mass is contained in smaller particles and half in larger particles
MMR	Mismatch repair; DNA-repair pathway correcting replication-associated mismatches and insertion/deletion loops
MN	Micronucleus/micronuclei; extranuclear body containing chromosomal material excluded from the main daughter nuclei
MRE	Metal response element; DNA regulatory sequence recognized by metal-responsive transcription factor 1
MT	Metallothionein; low-molecular-weight, cysteine-rich metal-binding protein involved in metal homeostasis and sequestration
MTF-1	Metal-responsive transcription factor 1; zinc-finger transcription factor controlling metal-responsive gene expression
NBUD	Nuclear bud; protrusion of nuclear material associated with the elimination or sequestration of amplified or incompletely resolved DNA
NER	Nucleotide excision repair; DNA-repair pathway that removes bulky and helix-distorting lesions
NHEJ	Non-homologous end joining; DNA double-strand-break repair pathway that rejoins DNA ends without an extended homologous template
NLRP3	NOD-, leucine-rich repeat- and pyrin domain-containing protein 3; sensor component of the NLRP3 inflammasome
NPB	Nucleoplasmic bridge; DNA-containing connection between daughter nuclei, commonly associated with dicentric chromosomes, telomere fusion, or unresolved DNA intermediates
NR	Not reported
NRF2	Nuclear factor erythroid 2-related factor 2; transcription factor coordinating antioxidant and electrophile-responsive gene expression
OECD	Organisation for Economic Co-operation and Development
OGG1	8-Oxoguanine DNA glycosylase 1; enzyme initiating the removal of 8-oxoguanine during base excision repair
OP	Oxidative potential; capacity of a particulate-matter sample to oxidize target molecules or generate redox-active responses under defined assay conditions
PGPR	Plant growth-promoting rhizobacteria; root- and rhizosphere-associated bacteria that promote plant growth and may modify metal mobility and uptake
PM	Particulate matter; heterogeneous suspension of solid particles and liquid droplets in air
PM_1_	Particulate matter with an aerodynamic diameter of ≤1 µm
PM_10_	Particulate matter with an aerodynamic diameter of ≤10 µm
PM_2.5_	Particulate matter with an aerodynamic diameter of ≤2.5 µm
PM_2.5–10_	Coarse particulate fraction with an aerodynamic diameter of >2.5 µm and ≤10 µm
PMF	Positive matrix factorization; receptor-modeling method that decomposes a concentration matrix into non-negative factor profiles and contributions
ROS	Reactive oxygen species; oxygen-derived reactive molecules involved in oxidative signaling and injury
RPA	Replication protein A; single-stranded-DNA-binding complex involved in DNA replication, repair, and ATR-dependent signaling
SCE	Sister chromatid exchange; reciprocal exchange of DNA between sister chromatids
SSB	Single-strand break; discontinuity affecting one strand of the DNA molecule
TDI	Tolerable daily intake; estimated amount that can be ingested daily over a lifetime without appreciable health risk
TET	Ten-eleven translocation dioxygenase
TF	Soil-to-plant transfer factor; empirical ratio between the metal concentration in a specified plant tissue and the corresponding concentration in soil
TLR4	Toll-like receptor 4; innate immune receptor involved in recognition and inflammatory signaling
TWI	Tolerable weekly intake; estimated amount that can be ingested weekly over a lifetime without appreciable health risk
WHO	World Health Organization
XRF	X-ray fluorescence; non-destructive or minimally destructive technique for elemental analysis of solid samples
